# Clinical decision support to *O*ptimize *C*are of patients with *A*trial *F*ibrillation or flutter in the *E*mergency department: protocol of a stepped-wedge cluster randomized pragmatic trial (O’CAFÉ trial)

**DOI:** 10.1186/s13063-023-07230-2

**Published:** 2023-03-31

**Authors:** David R. Vinson, Adina S. Rauchwerger, Chandu A. Karadi, Judy Shan, E. Margaret Warton, Jennifer Y. Zhang, Dustin W. Ballard, Dustin G. Mark, Erik R. Hofmann, Dale M. Cotton, Edward J. Durant, James S. Lin, Dana R. Sax, Luke S. Poth, Stephen H. Gamboa, Meena S. Ghiya, Mamata V. Kene, Anuradha Ganapathy, Patrick M. Whiteley, Sean C. Bouvet, Leon Babakhanian, Edward W. Kwok, Matthew D. Solomon, Alan S. Go, Mary E. Reed, on behalf of the Kaiser Permanente CREST Network

**Affiliations:** 1 The Permanente Medical Group, Oakland, CA USA; 2 Division of Research, Kaiser Permanente Northern California, Oakland, CA USA; 3 Department of Emergency Medicine, Kaiser Permanente Roseville Medical Center, Roseville, CA USA; 4 Department of Emergency Medicine, Kaiser Permanente San Jose Medical Center, San Jose, CA USA; 5 School of Medicine, University of California, San Francisco, San Francisco, CA USA; 6 Department of Emergency Medicine, Kaiser Permanente San Rafael Medical Center, San Rafael, CA USA; 7 Department of Emergency Medicine, Kaiser Permanente Oakland Medical Center, Oakland, CA USA; 8 Department of Emergency Medicine, Kaiser Permanente South Sacramento Medical Center, Sacramento, CA USA; 9 Department of Emergency Medicine, Kaiser Permanente Modesto Medical Center, Modesto, CA USA; 10 Department of Emergency Medicine, Kaiser Permanente Santa Clara Medical Center, Santa Clara, CA USA; 11 Department of Emergency Medicine, Kaiser Permanente Walnut Creek Medical Center, Walnut Creek, CA USA; 12 Department of Emergency Medicine, Kaiser Permanente San Francisco Medical Center, San Francisco, CA USA; 13 Department of Emergency Medicine, Kaiser Permanente South San Francisco Medical Center, San Francisco, CA USA; 14 Department of Emergency Medicine, Kaiser Permanente San Leandro Medical Center, San Leandro, CA USA; 15 Asolva, Pasadena, CA USA; 16 Department of Cardiology, Oakland Medical Center, Oakland, CA USA; 17 Departments of Epidemiology, Biostatistics, and Medicine, University of California, San Francisco, CA USA; 18 Department of Medicine, Stanford University, Palo Alto, CA USA

**Keywords:** Atrial fibrillation, Atrial flutter, Emergency medicine, Randomized trial, Cardioversion, Anticoagulants, Patient admission

## Abstract

**Background:**

Management of adults with atrial fibrillation (AF) or atrial flutter in the emergency department (ED) includes rate reduction, cardioversion, and stroke prevention. Different approaches to these components of care may lead to variation in frequency of hospitalization and stroke prevention actions, with significant implications for patient experience, cost of care, and risk of complications. Standardization using evidence-based recommendations could reduce variation in management, preventable hospitalizations, and stroke risk.

**Methods:**

We describe the rationale for our ED-based AF treatment recommendations. We also describe the development of an electronic clinical decision support system (CDSS) to deliver these recommendations to emergency physicians at the point of care. We implemented the CDSS at three pilot sites to assess feasibility and solicit user feedback. We will evaluate the impact of the CDSS on hospitalization and stroke prevention actions using a stepped-wedge cluster randomized pragmatic clinical trial across 13 community EDs in Northern California.

**Discussion:**

We hypothesize that the CDSS intervention will reduce hospitalization of adults with isolated AF or atrial flutter presenting to the ED and increase anticoagulation prescription in eligible patients at the time of ED discharge and within 30 days. If our hypotheses are confirmed, the treatment protocol and CDSS could be recommended to other EDs to improve management of adults with AF or atrial flutter.

**Trial registration:**

ClinicalTrials.gov NCT05009225.  Registered on 17 August 2021.

**Supplementary Information:**

The online version contains supplementary material available at 10.1186/s13063-023-07230-2.

## Introduction

### Background

Atrial fibrillation (AF) and atrial flutter are prevalent in the USA and are likely to escalate as the population continues to age. These atrial arrhythmias have a substantial impact on quality of life and patient health, increasing the risk for heart failure, thromboembolism, hospitalization, and death. The economic burden on the health care system is considerable [1, 2].

Patients with symptomatic AF and atrial flutter often present to the emergency department (ED) for treatment. There is no definitive evidence supporting optimal ED management of patients with AF and atrial flutter. Treatment strategies vary widely between countries, within countries, and within facilities [3–8]. Not all of this variation is warranted [9]. Implementation of professional society-based guidelines may help standardize care around best practices. But professional society-based guidelines for AF treatment vary in the amount of attention given to emergency medicine-related issues and offer variable recommendations for acute management [10–14].

Using recommendations from various clinical practice guidelines, as well as from primary studies and internal best practices, we created a set of recommendations for emergency medicine physicians in our integrated healthcare delivery system, addressing three leading aspects of ED care: (1) achieving sustained rate reduction for patients with rapid ventricular response; (2) optimizing cardioversion by increasing first-shock success or using suitable pharmacologic agents; (3) increasing implementation of stroke prevention actions in eligible patients being discharged home. By improving rate reduction and cardioversion, we sought to reduce hospitalization in patients with isolated AF or atrial flutter, at least in medical centers with higher hospitalization rates [15]. By promoting stroke prevention actions, we sought to increase the 30-day incidence of anticoagulation initiation for eligible patients.

With the goal of making our treatment recommendations readily available to physicians at the point of care, we designed a web-based clinical decision support system (CDSS), called RISTRA-AF (RISTRA stands for Risk Stratification). This decision support application, similar to prior RISTRA applications, is embedded within the ED navigator of the electronic health record (EHR) system of a large U.S. integrated health system [16, 17]. We first undertook a three-center pilot study to evaluate the feasibility and user response of the CDSS, which allowed us to improve RISTRA-AF. In late 2021, we began the O’CAFÉ trial, a stepped-wedge cluster randomized trial across 13 community EDs, the methods of which we describe here.

### Objectives

We have two primary aims:

1. To reduce initial hospitalization of adults (≥ 18 years) presenting to the ED with isolated AF or atrial flutter.
2. To increase the proportion of ED adult health plan members with primary AF or atrial flutter eligible for anticoagulation being discharged home who are prescribed anticoagulation either at the time of discharge or within the following 30 days.

### Hypotheses

1. We hypothesize that implementation of RISTRA-AF will reduce initial hospitalization for ED adults (≥ 18 years) with isolated AF and atrial flutter (Aim 1).
2. We hypothesize that implementation of RISTRA-AF will increase the proportion of ED adult health plan members eligible for anticoagulation initiation on discharge to home who are prescribed anticoagulation at the time of discharge or within the following 30 days (Aim 2).

### Trial design

The O’CAFÉ trial is a stepped-wedge cluster randomized pragmatic superiority trial across 13 EDs in a large, integrated healthcare delivery system in the USA. Trial EDs were selected by (a) having an on-site study champion (a clinical peer of the department and a co-investigator with the CREST research network (https://www.kpcrest.net); *n* = 16) and (b) having not already participated in the pilot study (*n* = 3). This design was selected over a parallel group design for three reasons [18, 19]: (1) The educational program of a staggered roll-out can be easier to implement than the alternative of a traditional parallel group design. With only one cluster launching each month, the principal investigator could co-present with site leads when introducing study material to their emergency and ancillary departments (i.e., adult hospital medicine and cardiology). This would be infeasible if multiple clusters launched simultaneously. (2) This design expands intervention exposure across all study EDs, which is desirable as the intervention is thought to be an improvement over usual care. (3) This approach maximizes power because the intervention effect is estimated not only by between-cluster comparisons but also by within-cluster comparisons.

We designed this as a pragmatic trial in which the intervention could be tested under conditions closer to usual care than ideal care [20].

**Fig. 1:**
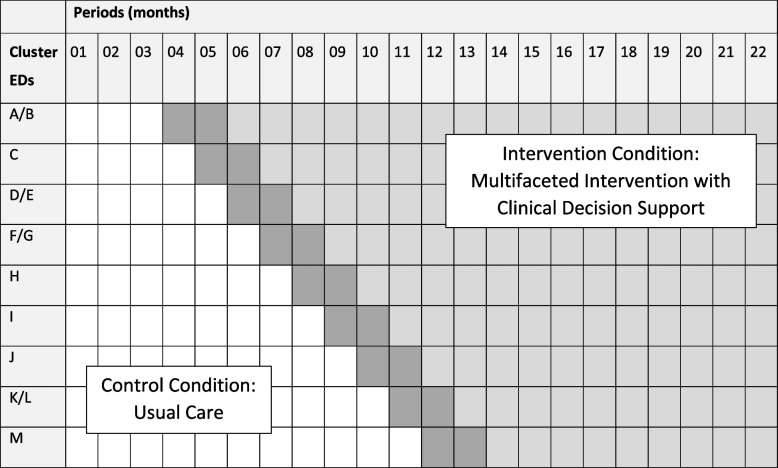
Time course over which 13 emergency departments (labeled A–M) crossed over from control condition to intervention condition. ED, emergency department. Unshaded cells depict periods of control condition; dark gray cells depict transition period (not analyzed); light gray cells depict periods of intervention condition

Among the 13 study EDs, eight functioned as four operational dyads, pairs of EDs, each served by one shared staff of emergency physicians. Keeping these EDs paired, we had nine study clusters, which the principal investigator allocated to one of nine sequences using a computer-generated randomization sequence. Site leads were not blinded to their launch month as they needed to schedule educational presentations. Physicians in study EDs could not be blinded to interventions; patients, however, were unaware of the trial. After an initial period of three months in which all clusters were in the control condition (July 2021 through September 2021), the intervention was implemented in one cluster per step at one-month intervals. The first two months of implementation were a transition period, which will not be analyzed. The staggered roll-out occurred over nine months (October 2021 through June 2022), after which 11 months followed during which all clusters were in the transition or intervention condition. The total study duration was planned for 22 months (Fig. 1), completing enrollment April 30, 2023. This manuscript complies with the SPIRIT 2013 checklist (Additional file 1) [21].

## Methods

### Study setting

The O’CAFÉ trial is being conducted in EDs of community medical centers in Kaiser Permanente (KP) Northern California, a large U.S. integrated health system of over 9000 physicians that currently provides comprehensive inpatient and outpatient care for more than 4.5 million members. Health plan members include over 33% of the population in areas served and are highly representative of the ethnic and socioeconomic diversity of the surrounding and statewide population [22]. Eighteen percent of patient encounters across the 21 EDs during 2021 were by non-members. Sixteen of the 21 EDs of KP Northern California have on-site emergency physicians who are embedded researchers and clinical investigators with the KP CREST Network https://www.kpcrest.net. They serve as site leads for pragmatic trials, providing necessary on-the-ground study promotion, physician education, and feedback among their peers [16]. Three of these 16 EDs had participated in the O’CAFÉ pilot study and were ineligible for the pragmatic trial. The remaining 13 EDs participated in the pragmatic trial. Their median ED volume for 2021 was 58,076 visits (IQR, 37,858 to 64,049; range 28,030 to 72,094).

KP Northern California is a learning health care system with a strategic delivery science agenda [23] and is supported by a comprehensive, integrated EHR that includes inpatient, outpatient, emergency, pharmacy, laboratory, and imaging data [24]. Six of the 13 study EDs participated to some degree in resident training. Patient care decisions were at the discretion of the treating physicians. No departmental policies or scripted pathways were in place for ED rate reduction or cardioversion prior to or concurrent with the intervention. In prior studies, we had observed significant inter-facility variation in ED AF management [15]. Treating physicians had access to the standard KP Northern California discharge order-set for AF-related stroke prevention, which during the study period recommended dabigatran, a direct oral anticoagulant, as first-line thromboprophylaxis for eligible patients. Outpatient anticoagulation with both warfarin and direct oral anticoagulants was managed closely by a pharmacy-led, telephone-based Anticoagulation Management Service [25]. All emergency physicians had around-the-clock access to on-call cardiology consultants. The KP Northern California Institutional Review Board approved the study with a waiver of informed consent given the minimal risk involved.

### Eligibility criteria of study participants and study patients

Study participants were all emergency physicians working in the 13 study EDs during the study period, all of whom were board-certified (or board-eligible) emergency physicians. A small proportion (< 5%) of emergency physicians were part-time moonlighters.

Study patients were adults (≥ 18 years) receiving care for primary AF or atrial flutter in a participating ED, regardless of whether the RISTRA-AF application was used. The designation of AF and atrial flutter as *primary* (vs secondary) was based on the diagnostic coding of the treating emergency physicians and included those with a primary diagnosis of AF or atrial flutter (International Classification of Diseases, tenth revision [ICD-10] codes I48.xx) as well as those whose primary diagnosis was palpitations (ICD-10 code R00.2) with a secondary diagnosis of AF or atrial flutter, confirmed by an ED 12-lead electrocardiogram interpretated by cardiology as diagnostic of AF or atrial flutter. We further specified AF and atrial flutter as *isolated *(as in aim 1) by excluding patients with co-existent diagnoses requiring emergency care or hospitalization (e.g., myocardial infarction, decompensated heart failure, severe sepsis) while blinded to ED treatments, as similar studies of ED AF hospitalization have done [26, 27]. Removing the influence of concomitant acute conditions other than AF or atrial flutter allowed the focus to remain on AF and atrial flutter management.

Patients were excluded from RISTRA-AF and from the O’CAFÉ trial for any of the following concurrent ED diagnoses: pregnancy, ST-elevation myocardial infarction, acute myo- or pericarditis, acute pneumonia, pulmonary embolism, shock (e.g., septic, hemorrhagic, cardiogenic), recent major thoracic trauma (< 48 h), thyroid storm, or acute toxidrome (e.g., sympathomimetic or anticholinergic). We did not include heart failure among our excluded co-diagnoses, as we wanted to provide treatment recommendations for patients with AF and atrial flutter and co-existing heart failure.

### Interventions

#### Multispecialty design team

We created our practice recommendations and the clinical decision support tool with a multispecialty team of co-investigators that included clinical researchers in emergency medicine, cardiology, critical care medicine, and information technology who collectively helped craft the content and design of our CDSS. We periodically consulted a project-specific advisory panel that included specialty leaders in cardiology, adult hospital medicine, primary care medicine, electrophysiology, hematology, endocrinology, and pharmacology.

#### Sources of recommendations

We reviewed published studies on the management of patients with AF and atrial flutter regarding rate reduction, cardioversion, and stroke prevention to identify best practices we could adopt or adapt for our physicians. We reviewed AF guidelines from cardiology societies in North America, Europe, Australia/New Zealand, and Asia [11, 13, 14, 28–35]. We also drew on expert panel consensus reports and best practices written for North American emergency physicians [12, 36].

#### Principles of management

We designed the general recommendations of RISTRA-AF to assist in the comprehensive management of most adults presenting to the ED with AF or atrial flutter. Treating patients whose AF might be secondary to or complicated by another acute condition (e.g., severe sepsis) often requires greater nuance [37, 38]. Here we advised physicians address and stabilize co-existent diagnoses before treating AF or atrial flutter.

As with all our clinical decision support tools, our recommendations were meant to be assistive, not directive; we sought to inform clinical decision-making, not constrain it. We enumerate our major recommendations with supporting rationales in Table 1.

**Table 1:** Management recommendations to improve care of ED patients with atrial fibrillation and flutter

Major recommendations in electronic clinical decision support application^a^	Rationale for recommendation
**1. Sustained rate reduction**	
Administer long-acting rate-reducing medications early in the ED encounter, either in addition to or in lieu of standard intravenous bolus medications	Medications with sustained effect on rapid ventricular response have been central to multifaceted ED interventions associated with reduced hospitalization of patients with isolated AF or atrial flutter [26, 27].
**2. Effective cardioversion**	
** 2A. Electrical**	
Start with maximal joules and consider manual pressure augmentation, especially for obese patients	These measures improve first-shock success and may reduce sedation duration and risk [13, 39–41].
** 2B. Pharmacologic**	
Consider efficiency in addition to effectiveness, safety, and ease of administration when selecting medications	For example, medications with a shorter time to effect, e.g., intravenous procainamide [42] (median 30–40 min), facilitate ED operational efficiencies, unlike intravenous amiodarone, which does not distinguish itself from placebo for 6–8 h [43].
**3. Stroke prevention**	
A. Identify patients at risk using auto-populating validated scoring system	Stroke risk stratification is the essential preparatory step for any subsequent stroke prevention action [13, 14, 29, 33].
B. Print risk-specific handout for eligible patients and review with patient and family at bedside	The handout helps initiate a shared decision-making conversation on stroke prevention [44] that can continue with outpatient physicians following discharge to home
C1. Initiate outpatient anticoagulation at the time of ED discharge to home	Oral anticoagulation with DOACs or warfarin significantly reduces ischemic stroke and death in patients with AF or atrial flutter. Prescription on ED discharge can be associated with higher long-term use than when prescribing is left to post-discharge outpatient care [45, 46].
C2. Or electronically consult the Anticoagulation Management Service to request they contact patients who want to learn more about stroke prevention before initiating anticoagulation	Following discharge to home, anticoagulation pharmacists can call eligible patients to provide in-depth education on benefits and risks of anticoagulation for stroke prevention [47, 48].
**4. Timely follow-up**	
a. Encourage or request close follow-up (< 7d) with outpatient physicians	Transferring care to outpatient physicians who can oversee longitudinal care of AF and atrial flutter and related conditions is key to long-term management success [14]. Moreover, follow-up of these patients within a week of discharge has been associated with a reduction in the rate of death and hospitalization within 1 year [49].

*AF* Atrial fibrillation, *DOAC* Direct oral anticoagulant, *ED* Emergency department

^a^RISTRA-AF also reminds physicians to inquire of their AF and atrial flutter patients about two dietary triggers: cold drink/food and alcohol (more below) [50, 51]

#### Clinical decision support system

We made our management recommendations readily accessible to emergency physicians by transposing them into an established web-based CDSS called RISTRA. We followed CDSS design principles that have been shown effective in our setting in earlier applications [52, 53]. The RISTRA system is currently used to provide point-of-care decision support to help emergency physicians in the management of adults with acute pulmonary embolism [16, 54], adults with chest pain [17], children 5 years of age or greater with acute abdominal pain [53, 55], febrile infants, and syncope/presyncope. RISTRA is accessed by a hyperlink button that was added to our ED navigator of the EHR (Epic, Verona, Wisconsin) and seamlessly fits within the flow of patient care [53, 56]. In what follows, we describe how a physician accesses and uses the AF application, explaining in more detail the recommendations we listed briefly in Table 1.

#### Self-directed activation of application with specific iPhone alert

In order to use the CDSS, the emergency physician voluntarily opens RISTRA, then selects RISTRA-AF from among the several condition-specific applications on offer (Additional file 2). We designed RISTRA access to be self-directed, as this feature is less intrusive than a best practice alert with a hard stop. There is no built-in prompt from within the EHR to identify AF or atrial flutter patients who are likely eligible for decision support. We do however provide an alert using a text-messaging tool that we have used successfully in other RISTRA studies [57]. We designed the RISTRA-AF alert to be highly specific, seeking to avoid false-positive activations that may increase the risk of alert fatigue [58]. The text message alert is triggered if an ED patient aged 18 years or greater is triaged with a study-specific chief complaint (AF or atrial flutter, palpitations, rapid or irregular heart rate, or fluttering heart) followed by a confirmatory 12-lead electrocardiogram. The alert arrives as a text message (Additional file 3) on the work smartphone of the emergency physician to whom the patient is assigned. Assignment occurs at ED triage in a round robin fashion [59]. Physicians could opt out of the smartphone alert.

#### Physician input on early screens to create accurate clinical portrait

The Start screen reviews eligibility, listing inclusion and exclusion criteria (Additional file 4). This screen also depicts the table of contents in a serial fashion in a progress bar across the top margin of the screen. Users may advance forward one section at a time and may jump back with one click to any prior section. If the physician reaches the module screen (see additional details below), the main workstation of the application, subsequent physicians who open RISTRA-AF on that same patient enter at the module screen. This is helpful when two or more physicians are both caring for the same patient, as when signing a patient over to another physician at the end of a shift or when sharing patient care duties with a resident physician. Most screens include a hyperlink to references, where we provide a list of publications that support management recommendations.

The next few RISTRA screens are interactive, collecting and confirming data that will inform upcoming decision support (Additional files 5, 6 and 7). These screens present to the physician patient-specific data drawn from structured fields in the EHR and provide opportunity for physician editing if needed [60]. This auto-populating feature is convenient for the physician, reducing the need to search through the medical record. Auto-populated variables include current anticoagulation, prior history of AF or atrial flutter, the date and results of the patient’s most recent echocardiogram, the date and result of the patient’s most recent thyroid-stimulating hormone test, as well as demographic and most of the medical history variables of the CHA_2_DS_2_-VASc score. Many of the auto-populated variables are provisional, subject to confirmation or correction by the treating physician. This user-editing capacity ensures a more accurate, complete portrait of the patient’s clinical characteristics, e.g., a more reliable CHA_2_DS_2_-VASc score.

**Fig. 2:**
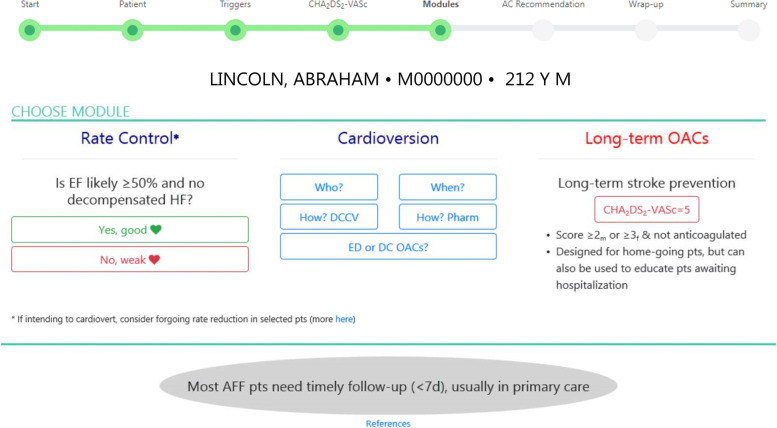
The module screen in RISTRA-AF. *AC* anticoagulant, *AFF* atrial fibrillation and flutter, *DC* discharge, *DCCV* direct current cardioversion, *ED* emergency department, *EF* ejection fraction, *HF* heart failure, *OAC* oral anticoagulant, *Pharm* pharmacologic cardioversion, *pt* patient

Variables that could not be auto-populated require physician entry, e.g., current cardiovascular clinical stability (with a definition provided in the hover text), atrial rhythm and duration of current episode (Additional file 5), select triggers (Additional file 6), and the vascular disease variable of the CHA_2_DS_2_-VASc score, particularly the aortic plaque component, which is not reliably identifiable in extracted EHR data using diagnosis codes (Additional file 7). Some decision support is provided in these early screens as hover text reminders about indications for additional testing, e.g., when to order echocardiography and a thyroid-stimulating hormone test (Additional file 5). The module screen is the hub of RISTRA-AF, with hyperlinks to recommendations on rate reduction, cardioversion, and stroke prevention (Fig. 2).

#### Common ingestion triggers

Though the ED is commonly the place where patients with acutely symptomatic AF and atrial flutter seek medical care, little attention in emergency medicine has been paid to the clinician’s role in helping patients identify and manage reversible triggers of paroxysmal AF and atrial flutter. To redress this oversight, we designed RISTRA-AF to prompt physicians to ask about two widespread ingestion triggers: cold drink/food and alcohol (Additional file 6). Cold drink and food can precipitate AF and atrial flutter within seconds or minutes of ingestion [61–64]. Some physicians are unaware of the causal connection between cold drink/food and AF and atrial flutter and have been known to dismiss their patient’s trigger claims [50]. Alcohol, on the other hand, is a well-known cause of AF and atrial flutter with “Holiday Heart” occurring during or following an alcohol binge [65, 66]. But AF can develop also within several hours of even one or two drinks [67]. Recent studies have elucidated the electrophysiologic mechanisms of this trigger [68].

When clinician-directed inquiries about these two triggers elicit a positive response, the stage is set for patient behavioral changes that may have remarkable benefits by decreasing the overall AF burden [51, 69]. Reducing recurrent events can reduce the attending symptoms, risk, and inconvenience of episodic AF and atrial flutter, as well as the costs incurred when the recurrence leads to missed work and the need for urgent medical care. We chose not to include coffee consumption among our list of triggers because the evidence does not support the commonly held belief that coffee triggers AF and atrial flutter [70, 71].

We are currently undertaking a survey study of patients with intermittent AF or atrial flutter who report a cold drink or food trigger to enlarge our understanding of this understudied phenomenon. We are reaching out to two groups of patients: those who were noted in RISTRA-AF by their treating emergency physicians to have a cold ingestion trigger (Additional file 6), as well as those who have emailed us in response to our case report or essay on “Cold Drink Heart”[50, 61, 63]. We will report the details of the survey study separately.

#### Rate reduction recommendations

Slowing rapid ventricular response is the most common treatment emergency physicians provide their patients with AF and atrial flutter. Intravenous medications, like the non-dihydropyridine calcium channel blocker diltiazem and the beta-adrenergic receptor blocker metoprolol, are effective heart rate-reducing medications (rate reducers) with a rapid onset [72–74]. Unfortunately, bolus doses of intravenous rate reducers can have a relatively short duration of action. The effect of a single bolus of intravenous diltiazem, for example, wanes after 1–3 h. If the rapid ventricular response returns, it can rebound higher than the initial rate. This may prompt another intravenous bolus of rate-reducing medication. If the rapid ventricular response again recurs or persists, a continuous infusion of diltiazem or esmolol may follow, which may result in admission to an observation unit or hospital ward for continued heart rate management.

**Fig. 3:**
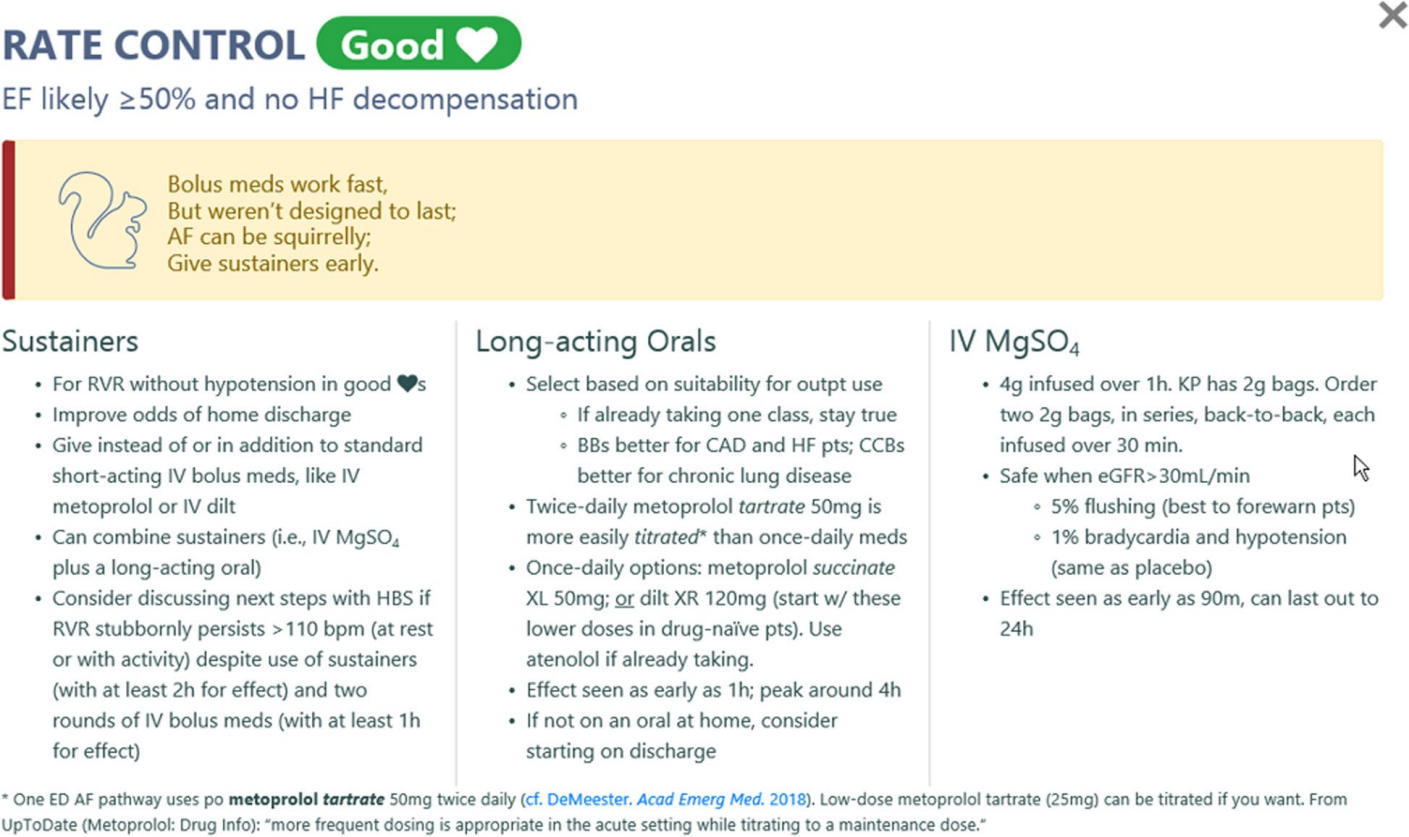
The rate control screen in RISTRA-AF for patients with a “good heart”. A good heart is defined as one with an ejection fraction greater than 50% (based on recent echocardiography or physician gestalt) and no clinical evidence of decompensated heart failure. BB, beta-blocker; bpm, beats per minute; CAD, coronary artery disease; CCB, calcium channel blocker; dilt, diltiazem; ED, emergency department; EF, ejection fraction; eGFR, estimated glomerular filtration rate; HBS, hospital-based specialist in internal medicine; HF, heart failure; IV, intravenous; KP, Kaiser Permanente; outpt, outpatient; Pharm, pharmacologic cardioversion; pt, patient; RVR, rapid ventricular response; w/, with

One strategy to avoid this common route to protracted care is the early administration of oral long-acting rate reducers, e.g., diltiazem XR or metoprolol tartrate. These can be given in addition to (or in lieu of) their intravenous counterparts [29]. The combination of shorter-acting intravenous medications with longer-acting medications has the advantage of providing both immediate and sustained rate-reducing effects. Several studies in different U.S. ED settings have found that treatment pathways encouraging early administration of a long-acting oral rate-reducing medication (with or without a concomitant intravenous rate reducer) decrease hospitalization of stable patients with isolated primary AF [26, 27]. Intravenous magnesium sulfate is another effective rate reducer, which can be helpful independent of a patient’s serum magnesium level [75–77]. Studies have shown continued effect lasting 12–24 h following initial magnesium sulfate administration [75, 77, 78]. Early administration of these “sustainers” (long-acting oral medications or intravenous magnesium sulfate) may reduce the need for hospitalization. We recommend “sustainers” only for normotensive patients with a “good heart,” defined as one with an ejection fraction greater than 50% (based on recent echocardiography or physician gestalt) and no clinical evidence of decompensated heart failure (Fig. 3).

Rate reduction in patients with hypotension, known left ventricular ejection fraction ≤ 50%, or decompensated heart failure is more challenging and warrants a different set of recommendations (Additional file 8). If the physician is intent on attempting electrical cardioversion in the ED and the stable patient is tolerating rapid ventricular response, we recommend against rate-reducing medications, as some evidence suggests they may reduce the effectiveness of electrical cardioversion (Additional file 9) [79]. This does not apply to patients who are to receive oral flecainide or propafenone, as they require a rate-reducing agent to block the atrioventricular node at least 30 min prior to cardioversion [28].

#### Cardioversion

Restoration of sinus rhythm is the most effective means of symptom resolution in patients with intermittent AF and atrial flutter and can be one component of a larger, long-term rhythm control strategy. Among ED patients, elective cardioversion is associated with reduced hospitalization and greater patient satisfaction [42, 80, 81]. RISTRA-AF provides recommendations about which ED patients may be candidates for elective and emergent cardioversion (Additional file 10) [13]. This information is accessible from the module screen via the link labeled “Who” (Fig. 2). Through the link labeled “When” (Fig. 2; Additional file 11), RISTRA-AF reminds physicians of the pros and cons to immediate attempted cardioversion compared with a short-term delay for those with symptomatic AF or atrial flutter of presumed recent-onset (< 48 h). The delayed approach is a “wait and see” approach that involves discharging the patient to home with a scheduled return visit at approximately 40 h post-symptom onset. We leave the timing debate (today vs tomorrow) open to accommodate physician and patient preference as well as varied local practice patterns [82–86]. The link “ED or DC OACs” (ED or discharge oral anticoagulation) summarizes recommendations from varied sources about which patients are thought safe to cardiovert without several weeks of preceding anticoagulation and which patients may benefit from anticoagulation following ED cardioversion (Additional file 12) [12, 13, 28, 87–90].

#### Electrical cardioversion: increasing first-shock success

**Fig. 4:**
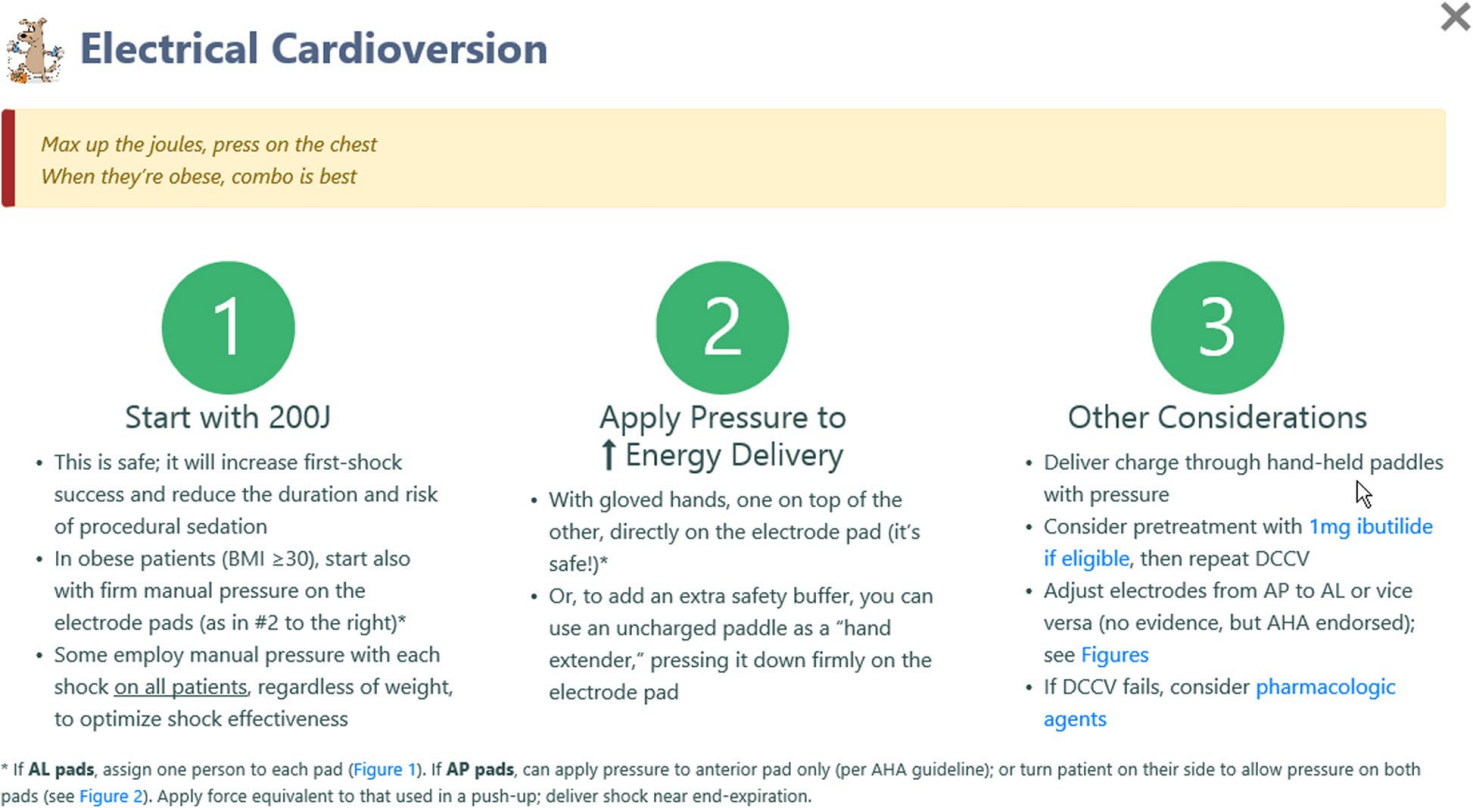
Electrical cardioversion screen in RISTRA-AF. AHA, American Heart Association; AL, anterior-lateral; AP, anterior–posterior; BMI, body mass index; DCCV, direct current cardioversion, max, maximize

When physicians elect to pursue ED cardioversion, we provide recommendations in RISTRA-AF to facilitate timely and effective sinus restoration (Fig. 4). With synchronized electrical cardioversion, we recommend maximizing joules to optimize first-shock success and limit sedation time and risk [28, 40, 41]. Most adverse events from electrical cardioversion are associated with sedation and not the procedure itself [91]. We recommend starting with maximal joules, which at present in our EDs is 200 (biphasic). If the first-shock fails, a second shock can be administered at 1 min. We recommend manual pressure augmentation to reduce transthoracic impedance, deliver more current to the heart and increase effectiveness of electrical cardioversion [39, 41, 92, 93]. Manual pressure augmentation has been shown to be safe for the proceduralist [39, 94]. It can be helpful for all patients, but more so for obese patients, who fail electrical cardioversion at twice the rate of non-obese patients [39].

If the first two shocks with maximal joules and manual pressure augmentation are unsuccessful, a priming dose of ibutilide (1 mg over 10 min) can be used in eligible patients (the criteria are spelled out in RISTRA-AF; cf. Additional file 13), followed by another attempt at electrical cardioversion. This has been shown to increase sinus restoration [28, 95]. In response to failed electrical cardioversion, RISTRA-AF follows U.S. guidelines in suggesting changing pad placement from anterior–posterior to anterior-lateral or vice versa [28]. One can also switch to pharmacologic approaches.

#### Time-efficient pharmacologic cardioversion

**Fig. 5:**
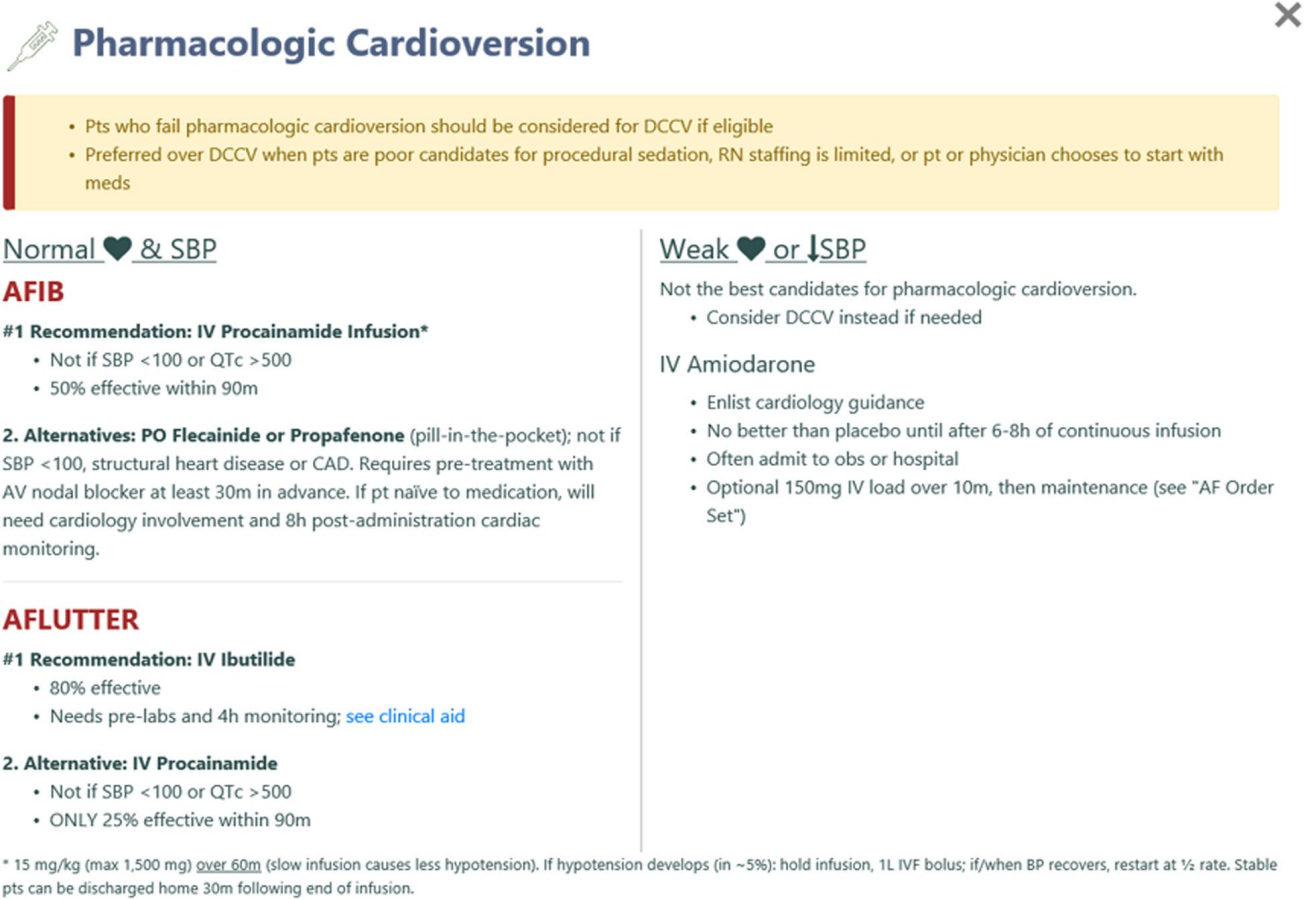
Pharmacologic cardioversion screen in RISTRA-AF. A weak heart is defined as one with an ejection fraction less than 50% (based on recent echocardiography or physician gestalt) or clinical evidence of decompensated heart failure. AFIB, atrial fibrillation; AFLUTTER, atrial flutter; AV, atrioventricular; BP, blood pressure; CAD, coronary artery disease; DCCV, direct current cardioversion; IV, intravenous; IVF, intravenous fluid; max, maximum; med, medication; obs, observation unit; pt, patient; pre-labs, pre-treatment laboratory testing; QTc, corrected QT interval; RN, registered nurse; SBP, systolic blood pressure

Pharmacologic cardioversion is less effective than an electrical approach [42]. However, it may be preferred when patients are poor sedation candidates or refuse electrical cardioversion, if ED nursing staff cannot easily support elective procedural sedation, or if physicians (or departments) prefer a two-step approach, starting with the less resource-intensive pharmacotherapy and reserving sedation and synchronized cardioversion for those who fail step 1 (Fig. 5) [96, 97].

Our medication recommendations are stratified by rhythm (AF vs atrial flutter), structural heart disease (good vs weak hearts, as defined above), and systolic blood pressure (Fig. 5). For normotensive patients without known structural heart disease, we suggest intravenous procainamide for several reasons: it is easy to administer, has a good safety profile, has a relatively rapid effect (over 50% at 90 m), does not require prolonged monitoring (unlike intravenous ibutilide in all patients [4h], oral flecainide and oral propafenone in drug-naïve patients [8h]), and has been well studied among unselected ED patients with presumed recent-onset AF (< 48 h) [42, 96]. Procainamide is the most common cardioversion medication used in Canadian EDs and the recommended drug-of-choice by the Canadian Association of Emergency Physicians for eligible ED patients with recent-onset AF [3, 12, 98].

Our second-line agents for pharmacologic cardioversion of hemodynamically stable ED patients with AF and good hearts are the oral agents propafenone and flecainide, famously used for the “pill-in-the-pocket” approach to rhythm control [99–102]. Though they may be more effective than procainamide in restoring sinus rhythm, Class Ic agents require pre-treatment with atrioventricular nodal blockers and, on first use, cardiology involvement and at least 8 h of cardiac monitoring, which in our system often involves admission to an observation or inpatient unit. What these medications gain in effectiveness, they lose in efficiency. If effective and safely tolerated in a monitored setting, these oral medications can subsequently be self-administered at home for the treatment of future paroxysmal AF episodes in select patients [100].

For normotensive patients with atrial flutter and no known structural heart disease, ibutilide is our drug-of-choice because of its effectiveness over intravenous procainamide (approximately 62 vs 25% at 90 m) [97, 103]. Intravenous ibutilide administration requires careful patient selection and protocol adherence to reduce the risk of polymorphic ventricular tachycardia, which is rare if ibutilide is properly used (Additional file 13) [97, 104]. The median time to effect of intravenous procainamide and intravenous ibutilide (approximately 30–40 m) contrasts sharply with intravenous amiodarone, which fails to reliably outperform placebo for 6–8 h [43]. This delay is not conducive to timely cardioversion and hampers departmental operational and resource efficiencies, often requiring admission to an observation unit or inpatient ward for administration [12]. Because of its limitations, intravenous amiodarone for ED patients with AF or atrial flutter is reserved for those with hypotension, left ventricular ejection fraction ≤ 50%, or decompensated heart failure, for whom intravenous procainamide and ibutilide, as well as oral flecainide and propafenone, are contraindicated. Because intravenous amiodarone recipients in our model of care are generally higher-risk patients, early cardiology consultation and inpatient monitoring are prudent to personalize safe management.

#### Stroke prevention

One of the most serious complications of AF and atrial flutter is ischemic stroke, which can be significantly disabling, if not fatal. Fortunately, thromboprophylaxis can reduce stroke risk by two-thirds and mortality by 25% [13, 28, 105]. Stroke prevention is a critical component of AF and atrial flutter management in all society guidelines [14, 106]. The ED provides an important opportunity to identify patients who meet criteria for anticoagulation, and ED care may serve as a sentinel moment for behavioral change [46, 107–109]. Initiating stroke prevention therapy at the time of ED discharge to home has been shown to be safe and associated with a mortality reduction [110]. Yet emergency physicians often under-prescribe anticoagulation on discharge of eligible patients with AF and atrial flutter [107, 108, 111]. In some health systems, patients interested in starting anticoagulation who receive a prescription at the time of ED discharge are more likely than their non-treated counterparts to be on anticoagulation one year later [112]. Several ED studies have used clinical decision support tools to increase ED prescribing of oral anticoagulants in eligible AF patients on discharge to home [113, 114].

However, some have debated whether the initiation of anticoagulation at discharge for home-going patients falls within the scope of ED care [115]. What cannot be debated is the value of identifying at-risk patients with AF and atrial flutter and informing them that stroke prevention is an important topic worth exploring with their outpatient physicians. Even a brief discussion on stroke prevention with an emergency physician may move eligible patients one step closer towards anticoagulation.

The CHA_2_DS_2_-VASc score is currently recommended in various society guidelines for stroke risk stratificiation [13, 28]. We opted to use it to identify patients at sufficient stroke risk to warrant anticoagulation, despite its significant shortcomings [116, 117]. To make the CHA_2_DS_2_-VASc score easier to use, we auto-populated it in RISTRA-AF by drawing in comorbidities from the EHR Problem List, as we have done with other clinical applications [17, 60]. All patients in RISTRA-AF receive a CHA_2_DS_2_-VASc calculation unless they have a stroke-prone condition in which anticoagulation is indicated regardless of their risk score: moderate-to-severe mitral stenosis, mechanical valve, or hypertrophic cardiomyopathy (Additional file 7). In these higher-risk patients not currently on anticoagulation, we recommend a consult to the pharmacy-led telephone-based Anticoagulation Management Service.

If the patient has an elevated CHA_2_DS_2_-VASc score (≥ 2 in men and ≥ 3 in women), is not currently taking an anticoagulant, and will be discharged to home, we recommend they receive one or more of the following stroke prevention actions (Additional file 14): (1) a risk-specific educational handout, reviewed at the bedside with the treating physician in a shared decision-making conversation (Additional file 15). The handout is designed to be taken home as part of the patient’s discharge instructions and can be used to facilitate discussion with family and with their outpatient physician; (2) if patients express interest in learning more about the benefits and risks of stroke prevention, the emergency physician can send an electronic consult to the Anticoagulation Management Service, which will contact eligible patients to discuss treatment options; (3) a 30-day prescription of an oral anticoagulant. Currently in our health system, dabigatran is the initially recommended anticoagulant for at-risk patients, if eligible. In RISTRA-AF, we provide guidance on dosing and contraindications (Additional file 14) and link the physician to a patient handout from the health system on the medication. If the physician wants to explore alternative anticoagulants, we provide links to internal resources on how to tailor the anticoagulant choice for patients with AF or atrial flutter.

Some emergency medicine pathways identify patients with AF or atrial flutter who are eligible for anticoagulation by using a high predicted stroke risk combined with a low estimated bleed risk, e.g., the HAS-BLED score [118]. We include on the anticoagulation screen a link to both the HAS-BLED score as well as a summary of how it was designed to be used (Additional files 16 and 17). The fundamental purpose of HAS-BLED is to draw attention to reversible risk factors that need correcting rather than to exclude patients from being recommended anticoagulation if they are at increased risk for ischemic stroke; patients with a higher HAS-BLED score require more careful review and closer monitoring by their outpatient care team [119].

#### Follow-up after ED discharge to home

It is critical to patient care and outcomes that emergency physicians transfer care to outpatient physicians who can continue to manage rhythm-related symptoms via rate or rhythm control and to refer for cardiology management as needed, e.g., for complex cases or procedural intervention like elective outpatient cardioversion or ablation. An equally important component of ongoing primary care management is to proactively manage cardiovascular risk factors and comorbidities such as obesity, hypertension, and diabetes [14, 29, 120, 121]. We recommend that patients with AF or atrial flutter receive timely outpatient follow-up (< 7 days) (Fig. 2). Some multidisciplinary ED treatment pathways for AF and atrial flutter create a new, dedicated outpatient clinic to facilitate post-ED follow-up [114, 122, 123]. Given our integrated health care delivery framework, health plan members have primary care physicians with whom timely follow-up is readily available (and those physicians have access to the same integrated EHR used in our EDs), so the creation of a specific AF clinic for discharged ED patients was unnecessary.

#### Wrap-up and summary

RISTRA-AF provides physicians an efficient way to document a structured summary of their AF-related ED management using the wrap-up screen (Additional file 18). This requires physician input about elements of ED care that we use to build a templated summary paragraph that can be copied from RISTRA-AF for pasting into the ED note of the EHR (Additional file 19).

#### Outcomes

The primary outcome for aim 1 is hospitalization among those with isolated AF or atrial flutter [26, 27]. This includes admission to the inpatient setting and to outpatient observation units. We selected this broad definition to distinguish hospitalization from discharge to home directly from the ED. We will undertake a sensitivity analysis using a stricter definition of hospitalization, which includes only admission to the inpatient setting, to accommodate inter-facility variation in outpatient observation practices. We will also undertake a sensitivity analysis evaluating hospitalization in the larger cohort of patients who were coded as having primary AF or atrial flutter, whether the atrial arrhythmia was isolated or not. This will allow evaluation of possible biases in coding practices and assignment of isolated status.

Secondary outcomes for aim 1 include (a) discharge to home < 24 h of ED registration; (b) total length of stay in the ED and hospital; and (c) ED administration of a long-acting rate-reducing medication among patients who received any rate-reducing medication, oral or intravenous, short- or long-acting. Long-acting rate-reducing medications include oral diltiazem XR, metoprolol tartrate, metoprolol succinate, and atenolol and intravenous magnesium sulfate, 2 g or more. We will undertake a sensitivity analysis in which only 4 g or more of intravenous magnesium sulfate will count as a long-acting rate-reducing medication, as recommended in RISTRA-AF. We are not including amiodarone among our rate-reducing medications because amiodarone can also be used for cardioversion, and we cannot readily distinguish the two indications. Another secondary outcome for aim 1 is administration of continuous IV infusion of diltiazem or esmolol, which may be reduced in patients receiving early long-acting rate-reducing medications.

The primary outcome for aim 2 is anticoagulation initiation in eligible patients with AF or atrial flutter at the time of ED discharge to home or within the following 30 days. Eligibility includes an elevated CHA_2_DS_2_-VASc score (≥ 2 in men and ≥ 3 in women) in health plan members not currently taking anticoagulants who are being discharged to home directly from the ED. Current anticoagulation use is defined using EHR data. A patient is considered to be taking oral anticoagulation if (a) any oral anticoagulation prescription was filled in 45 days prior to the index encounter, (b) the supply of a filled prescription would include the index encounter date, or (c) active use of an oral anticoagulant was documented in the medication review during the index encounter or during the 30 days prior. A secondary outcome of aim 2 is electronic consultation of the Anticoagulant Management Service, independent of anticoagulation initiation in eligible patients (defined above).

We will measure use of RISTRA-AF but have not made it a major outcome. Use of the tool may not correlate strongly with use of the management principles that the decision support tool recommends [124]. For example, a physician may use RISTRA-AF to learn that the most effective means of electrically cardioverting an obese patient involves starting with maximal joules combined with manual pressure augmentation. This management principle is easily remembered and may be employed in future cases without recourse to RISTRA-AF. Frequency of tool use would serve as a poor indicator of frequency of optimal electrical cardioversion technique. We observed a similar pattern in prior decision support research on site-of-care decision-making for ED patients with acute pulmonary embolism. Over the course of the 4 years following the initial implementation of the decision support tool, the frequency of safe outpatient management of ED patients with low-risk pulmonary embolism increased though tool use decreased [54]. These two cases (electrical cardioversion of AF and outpatient management of low-risk pulmonary embolism) illustrate why use of our RISTRA tools is optional. We provide these tools to aid clinicians with management decisions if they feel assistance is needed. Use of the CDSS is not mandatory.

#### Pilot study

We undertook an eight-month pilot study at three geographically-affiliated KP Northern California medical centers, two where the principal investigator worked and a third nearby medical center with whom emergency physicians were sometimes shared. We launched the pilot by providing a series of educational resources to the emergency physicians and adult hospitalists identical to what we used for study launch (see below). We asked the pilot study physicians to use RISTRA-AF and send us questions and comments about the user interface and the educational and guidance content. We received much helpful input and used the feedback to improve the content and presentation of RISTRA-AF as well as the accompanying educational materials (e.g., our introductory presentation). Major changes made to RISTRA-AF during the pilot phase included the following examples: We expanded our criteria of excluded co-diagnoses to include pneumonia, acute myocarditis, and acute pericarditis; we clarified the timing of post-ED follow-up as within 7 days; we reworded the hover text explaining the indications and instructions for an electronic consult to the Anticoagulation Management Service; we added a hyperlink to facilitate placement of the electronic consult; we created a “training mode” for physicians to explore RISTRA-AF on any current ED patient (regardless of RISTRA eligibility) to gain familiarity with the tool without patient enrollment; we changed formatting and wording in several places to improve clarity; we expanded the references.

Additionally, we undertook manual chart review of randomly selected cases to validate our computerized algorithm for case ascertainment (see above on eligibility criteria). Among 100 cases identified as study eligible, 98 were confirmed to be eligible. Among 50 cases with AF who were identified as having exclusion criteria, the algorithm was correct in 49 cases.

#### Educational outreach prior to launching RISTRA-AF at each study site for the pragmatic clinical trial

Our introductory educational program for physicians included three elements, undertaken in a serial fashion: (1) The first was a roughly 30-min teaching presentation given to physicians during a regularly scheduled department-wide meeting. Introductory slides were sent to the department to be available for non-attendees and were accessible via a link underneath the RISTRA-AF entry button on the landing page (Additional file 2). The presentation included instructions on how to access RISTRA-AF, the nature of its content, and the rationale and evidence base for its principal recommendations (Table 1). (2) The second educational resource was access to RISTRA-AF using “training mode,” an option available on the first screen of the application (Additional file 4). Training mode allows physicians to test drive the application on any current ED patient, whether or not the patient is eligible for the tool. This allows the physician to become familiar with the content, recommendations, hover texts, and links without formally registering the patient in the study. (3) After completing the initial training and testing the application, physicians could take a short quiz (called a training survey) to test their knowledge of RISTRA-AF content. If they failed to pass with a score of 85% or higher, we sent them “hints” (i.e., the questions with the answers highlighted) and asked them to retake the quiz. Those who passed received up to three small gift cards, one for each of their first three completed enrollments (completed = reaching the summary screen). A similar incentive model had been used in prior RISTRA studies [16].

We also included the local cardiology and adult hospitalist departments in our launch-related education. We sent a one-page executive summary to the local chiefs of both departments, along with the introductory slide deck, which they sent to their respective physicians to inform them of the application and our clinical trial. We offered to give to their departments a presentation if they wished; some invited us to explain our study at their department meeting. We also informed the ED’s local nurses and inpatient pharmacists of the study. The inpatient pharmacists worked with us to be sure each study ED had the long-acting rate-reducing medications in their ED’s automated medication dispensing system for ready access.

#### Facility-specific monthly engagement by site leads

Local site leads provide their EDs with monthly emails. The content varied month by month, but routinely included commendation to recent local RISTRA-AF users, highlights of overall study progress, and “test your knowledge” questions to keep the AF and atrial flutter education going that began in the training survey. Six months following launch at a study site, site leads presented at an ED meeting a brief overview of the application and fielded questions from their peers. Starting at month 12 (June 2022; Fig. 1), metrics were incorporated into the monthly emails that included three intra-facility comparisons: use of long-acting rate reduction medications, anticoagulation prescribing at time of ED discharge to home, and anticoagulation prescribing in the 30 days following discharge. Each graphic included metrics of the three leading EDs as well as the ED in question (see Additional file 20 for an example).

#### Data collection and management

The majority of data in the O’CAFÉ trial were extracted from the EHR, based on structured fields populated by operational processes. Likewise, most data in RISTRA-AF were auto-populated from the EHR, based on prescribed data lists or range limits. Data entry, on the other hand, occurred by physicians only during RISTRA-AF use and only for select variables (see “Physician Input” above). These data, e.g., duration of symptoms, were used in the trial only for descriptive purposes.

Missing data were rare. Aim 1, for example, addresses hospitalization and administration of long-acting rate-reducing medications, ED process of care measures available on all study patients, independent of health plan membership. Aim 2, however, requires a complete medical profile to calculate a CHA_2_DS_2_-VASc score and 30-day follow-up to identify anticoagulation initiation. For these reasons, aim 2 was limited to study patients with health plan membership. In cases where missing data pertain, we either excluded patients with missing data from the specific analysis, used multiple imputation to estimate a value for the individuals with missing data, or created indicator variables for missing values in models to determine if missingness was predictive of outcomes.

Protected health information was stored in password-protected encrypted electronic files on protected servers. Only aggregate, deidentified data will be shared outside the research team. All personal identifiers will be destroyed at the end of the study. Only the study’s analyst and research scientist (EMW and MER, respectively) will have access to the final trial dataset.

#### Analytics and sample size

The aim of the O’CAFÉ trial was to determine whether the intervention reduces hospitalization among eligible ED patients with isolated AF or atrial flutter. We also aimed to determine if the intervention was associated with increased initiation of oral anticoagulants among eligible patients at high stroke risk. To this end, we designed the trial as a superiority trial to determine if intervention sites had better outcomes than control sites.

Analysis of RISTRA-AF effectiveness will be based on comparison of intervention and control groups according to the stepped-wedge cluster randomized pragmatic trial design. All analyses for this stepped-wedge group-randomized trial will be approached using mixed model regression methods. As this is a group-randomized trial and all groups will receive the intervention, all analyses will be done as intent-to-treat. Because the CDSS is available to all ED physicians at each study site and training is given at the site level as part of the intervention initiation, all patients at a given facility will be seen by a physician with access to and knowledge of the CDSS and therefore meet the study definition of “treated.” We will not depend upon CDSS utilization data to identify patients who received CDSS-enhanced care, because as physicians became more familiar with the CDSS content and internalized it, they would be less likely to use the tool during a patient encounter but still deliver care in accordance with the CDSS recommendations. Outcome and predictor measures were derived from the EHR, based on operational processes. We will examine within- and between-cluster correlation over time to elucidate possible correlation structures, including possible time decay in the correlation over time. While intraclass correlation and the number of repeated individuals in our cohort are expected to be low based on pilot study data, we will describe the intraclass correlation and churn rates over time and by cluster. We will use descriptive statistics to examine outcome trends over time overall, by cluster and by intervention status. Following the methods for open cohort stepped-wedge designs with binary outcomes outlined by Li et. al [125], we will use mixed models to allow for clustering with appropriate correlation structures, adjusting for time effects, RISTRA-AF status, and possibly hospital-level fixed effects.

We estimate that our stepped-wedge design (with nine clusters and 10 steps) will include approximately 3420 adult ED encounters with primary and 972 (30%) with isolated AF or atrial flutter during the 10-month roll-out period. Based on pilot data, we expect at least 567 patients in the usual care condition and 460 patients in the intervention condition with isolated AF or atrial flutter during the 10-month roll-out period. Using preliminary data at the pilot sites and the trial sites, baseline initial hospitalization rate was 26.6%. We estimate a minimally detectable 8% absolute difference in initial hospitalization rate (Aim 1) at a level of 90% power and a 2-sided test at the 2.5% significance level.

We estimated the minimum number of clusters needed to achieve 90% power based on pilot data using the National Institute of Health Stepped-wedge Group Randomized Trial Calculator [126]. We present our most conservative estimates here. For the hospitalization outcome, we assume an average of 11 eligible encounters per cluster, intraclass correlation of 0.01, the cluster autocorrelation of 0.47, and the individual autocorrelation of 0.9 with a discrete-time decay, a churn rate of 0.942 and adjustment for one cluster-level variable (annual ED census) with R [2] of 0.07. We would need only three clusters to see a decrease of 8% in hospitalization rates at 90% power, so we believe that we have adequate power in this analysis, given data from nine clusters over the course of the trial.

Given that only 18% of ED encounters are eligible for stroke prevention action (discharged to home, current KP member, not currently or recently taking oral anticoagulants, and at high risk for stroke), the overall numbers of eligible encounters for the stroke prevention-related outcomes are much smaller. For the primary Aim 2 outcome (any prescription ordered for oral anticoagulation medications within 30 days of the index visit), power is still adequate in this study design to identify changes in rates of prescriptions ordered as small as 5% in the eligible subgroup. Based on pilot data and assuming an average of seven eligible encounters per cluster, intraclass correlation of 0.006, the cluster autocorrelation of 0.356, and the individual autocorrelation coefficient of zero with a discrete-time decay, and a churn rate of 0.984 with no adjustments for cluster-level variables, our nine-cluster design will allow us to identify a 4.9% change in rates of anticoagulant prescription with 80% power.

We anticipate wide variation in practice patterns across our EDs, as we have seen in the management of other conditions [54, 127, 128]. Some EDs might start the trial further from their optimal performance level than others. These EDs may have more potential for practice improvement than others and more to gain from the intervention. To account for this, we will also report facility-specific changes from pre- to post-intervention, anticipating a larger impact at facilities whose pre-intervention practices were in the lower tertile.

Given the many variables we are collecting during this trial, we also will be able to address other important clinical questions. For example: What is the association of short-acting oral rate-reduction medications (e.g., diltiazem 30 mg) with hospitalization? Is timing of administration of long-acting rate-reducing medications (e.g., early vs late in the ED course) associated with ED length of stay and hospitalization? How does hospitalization prevalence compare between those receiving different doses of intravenous magnesium sulfate? What are the prevalence and effects of administering both non-dihydropyridine calcium channel blockers and beta-blockers? Was the trial intervention associated with a change in cardioversion prevalence and success and in selection of cardioversion agents (e.g., electrical vs pharmacologic; procainamide vs ibutilide) for AF and atrial flutter? What is the association of stroke prevention actions in the ED with the short- and long-term incidence of ischemic stroke and death among patients eligible for anticoagulation on ED discharge to home? Was the trial intervention associated with other measures of patient care recommended by RISTRA-AF, e.g., ordering of thyroid-stimulating hormone and echocardiography testing when indicated?

#### Dissemination policy

We will use authorship eligibility criteria from the International Committee of Medical Journal Editors. We intend to disseminate our results within the medical group and health system. We also will submit abstracts for presentation at professional society meetings and submit manuscripts to clinical journals for peer review.

## Discussion

We are implementing a stepped-wedge cluster randomized pragmatic trial, O’CAFÉ, to evaluate the effect of RISTRA-AF, an electronic CDSS integrated into the EHR, to improve care of ED patients with AF or atrial flutter. The tool addresses comprehensive ED management, which includes administration of long-acting rate-reducing medications, best practices for more effective, time-efficient cardioversion, and identification of and recommended actions for AF and atrial flutter patients at increased predicted risk for ischemic stroke (Table 1). We hypothesize that the intervention will be associated with reduced initial hospitalization (Aim 1) and increased 30-day anticoagulation initiation (Aim 2).

There have been few randomized trials in the ED implementing best practices to improve the care of patients with AF or atrial flutter. RAFF-3 was such a trial, a stepped-wedged cluster randomized trial conducted across 11 EDs in Canada [98]. Investigators implemented the 2021 Acute AF/Flutter Best Practices checklist from the Canadian Association of Emergency Physicians [12]. The trial’s primary aim was to reduce ED length of stay by promoting ED cardioversion over a rate-reduction strategy. They also sought to increase anticoagulation initiation at the time of discharge in eligible patients. The study population included only hemodynamically stable patients presenting with an episode of “acute” AF or atrial flutter of at least 3 h duration, where symptoms required ED management by rhythm or rate control. Patients with permanent AF or atrial flutter were excluded. Over 90% of enrolled patients had presumed recent-onset AF or atrial flutter < 48 h in duration. The intervention increased the proportion of patients undergoing attempted cardioversion (from 67 to 85%), reduced the use of rate control measures (from 54 to 38%), increased the proportion of patients with sinus restoration (from 76 to 86%), and reduced ED length of stay by 21% [98]. There was no improvement in new anticoagulation prescriptions at the time of ED discharge.

The O’CAFÉ trial differs in several ways. We included a broader population of patients with AF and atrial flutter; we did not limit eligibility by hemodynamic stability or arrhythmia duration, for we sought to address care for a wide spectrum of patients with primary AF and atrial flutter. Our strategy of recommendations was also different. Unlike the RAFF-3 trial, we did not seek to increase cardioversion and decrease rate control strategies, even for patients with presumed recent-onset AF or atrial flutter. We let the treating physician decide whether rhythm or rate control was indicated, only reminding them of when different strategies might be appropriate. Our rate and rhythm recommendations focused on ways of optimizing the chosen intervention. For the physician pursuing cardioversion, for example, we recommend methods to increase first-shock success. For the physician pursuing rate reduction, we recommend long-acting medications that facilitate sustained rate reduction and have been associated with reduced hospitalization.

Other ED-based trials have addressed more specific questions of AF management among subpopulations of ED AF patients, e.g., rapidity of rate reduction (intravenous diltiazem vs intravenous metoprolol) [72, 73], method of cardioversion (pharmacologic vs electrical) [129, 130], medication of cardioversion (e.g., intravenous vernakalant vs intravenous ibutilide) [131], timing of cardioversion (early vs delayed) [82, 83], and location of acute management (observation unit vs inpatient ward) [132].

Our decision support application did not have only treating physicians in view. We were equally mindful of our patients. Reducing hospitalization, one of our study aims, is an outcome valued by patients [133, 134]. Symptom reduction is also highly prized by patients. We pursued this patient-centered outcome by seeking to improve sustained rate reduction and increase the success of cardioversion, both of which reduce symptoms caused by the rapid ventricular response common in patients with AF and atrial flutter.

In sum, we describe the design of a CDSS to aid in the comprehensive management of ED patients with AF or atrial flutter, its pilot in three EDs, and its implementation using a stepped-wedge cluster randomized trial across 13 EDs of an integrated care system in northern California. This trial sought to improve uptake of best practices, improving measures of rate reduction, cardioversion, and stroke prevention. In particular, the study aimed to reduce hospitalization and increase anticoagulation prescribing for eligible patients. We will publish our results upon study completion.

## Trial status

Protocol version 10 (13 February 2023); enrollment (recruitment) start date: 1 October 2022; projected enrollment (recruitment) completion date: 30 April 2023.

## Supplementary Information

**Additional file 1. **SPIRIT 2013 Checklist.

**Additional file 2. **Landing page of CREST decision-support applications.

**Additional file 3. **Smartphone alert

**Additional file 4. **Start screen.

**Additional file 5. **Patient screen.

**Additional file 6. **Trigger screen with explanation paragraph.

**Additional file 7. **CHA_2_DS_2_-VASc Screen.

**Additional file 8. **Rate control for a weak heart.

**Additional file 9. **Rate control if cardioverting.

**Additional file 10. **Eligibility for ED cardioversion.

**Additional file 11. **Debate over timing of ED elective cardioversion.

**Additional file 12. **Anticoagulation when cardioverting.

**Additional file 13. **Ibutilide decision aid.

**Additional file 14. **Anticoagulation recommendations for patients with high estimated annual risk for ischemic stroke.

**Additional file 15. **Patient handout on stroke risk.

**Additional file 16. **About HAS-BLED.

**Additional file 17. **HAS-BLED score and risk.

**Additional file 18. **Wrap-up screen

**Additional file 19. **Summary to paste into physician charting.

**Additional file 20. **Example of facility-specific monthly graphic on sustainers use.

## Data Availability

Not applicable.

## Abbreviations

AF: Atrial fibrillation
CDSS: Clinical decision support system
ED: Emergency department
EHR: Electronic health record
O’CAFÉ trial: Clinical decision support to *O*ptimize *C*are of patients with *A*trial *F*ibrillation or flutter in the *E*mergency department trial
RISTRA: Risk stratification application
