# Supplementary material for: Clinical decision support to Optimize Care of patients with Atrial Fibrillation or flutter in the Emergency department: protocol of a stepped-wedge cluster randomized pragmatic trial (O’CAFÉ trial)
Source: Trials. 2023 Mar 31;24:246. doi: 10.1186/s13063-023-07230-2 (PMC10064588; doi:10.1186/s13063-023-07230-2)
Supplement: Supplementary file 2 — Additional file 2. Landing page of CREST decision-support applications. [file 13063_2023_7230_MOESM2_ESM.pdf]

Additional file 2:
Landing Page

RISTRA
RISK STRATIFICATION

Asolva

CREST NETWORK

A RESEARCH PORTFOLIO OF CREST NETWORK

ACUTE CORONARY SYNDROME

FAQ

PULMONARY EMBOLISM

PEDIATRIC ABD PAIN

FAQ

AFIB or FLUTTER

- Executive Summary (1 page)
- Introductory Slides
- Training Survey (10 min)

INFANT FEVER

- FAQ (Oct 2021)
- Roseville Protocol Study (Dec 2020)
- AAP Guidelines (Jul 2021)
- CA FIRST 2.0 Slides/Webinar/Algorithms

SYNCOPE

- Executive Summary (1 page)
- Introductory Slides
- Training Survey (5 min)

www.kpcrest.net

RISTRA tools are components of ongoing research. As always, use your clinical judgment.
