# Supplementary figures and images for: Clinical decision support to Optimize Care of patients with Atrial Fibrillation or flutter in the Emergency department: protocol of a stepped-wedge cluster randomized pragmatic trial (O’CAFÉ trial)
Source: Trials. 2023 Mar 31;24:246. doi: 10.1186/s13063-023-07230-2 (PMC10064588; doi:10.1186/s13063-023-07230-2)

Additional file 3:  
Smartphone Alert

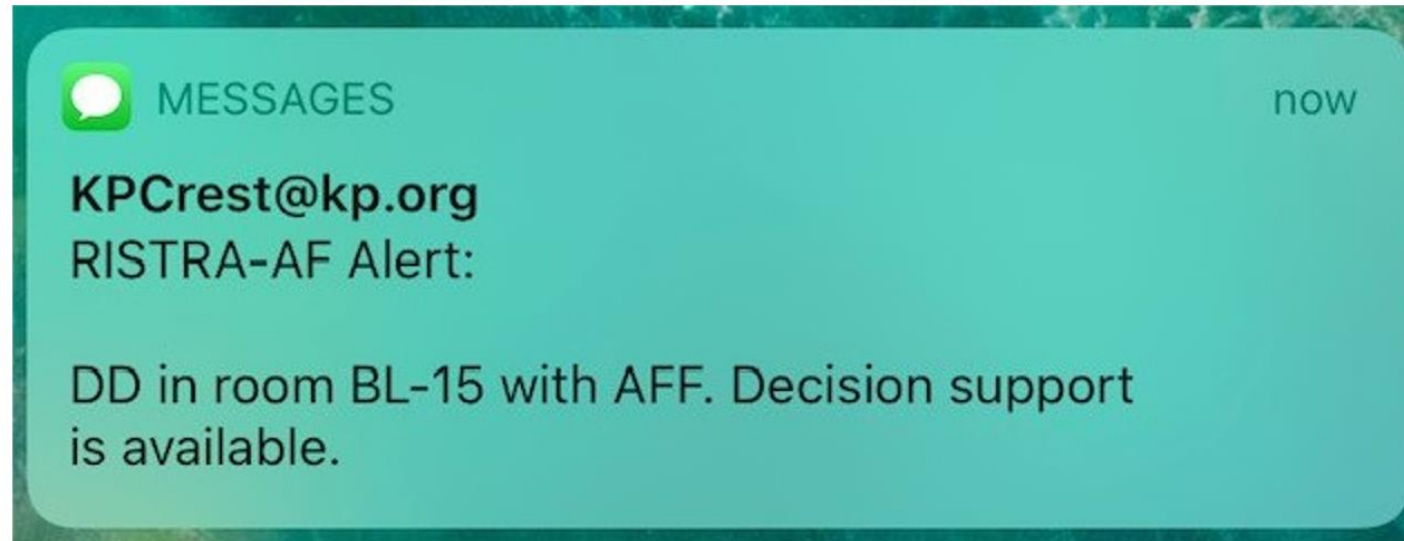

Supplement: Supplementary file 3 — Additional file 3. Smartphone alert [file 13063_2023_7230_MOESM3_ESM.pdf]

Additional file 20:  
Example of facility-specific monthly  
graphic on sustainers use

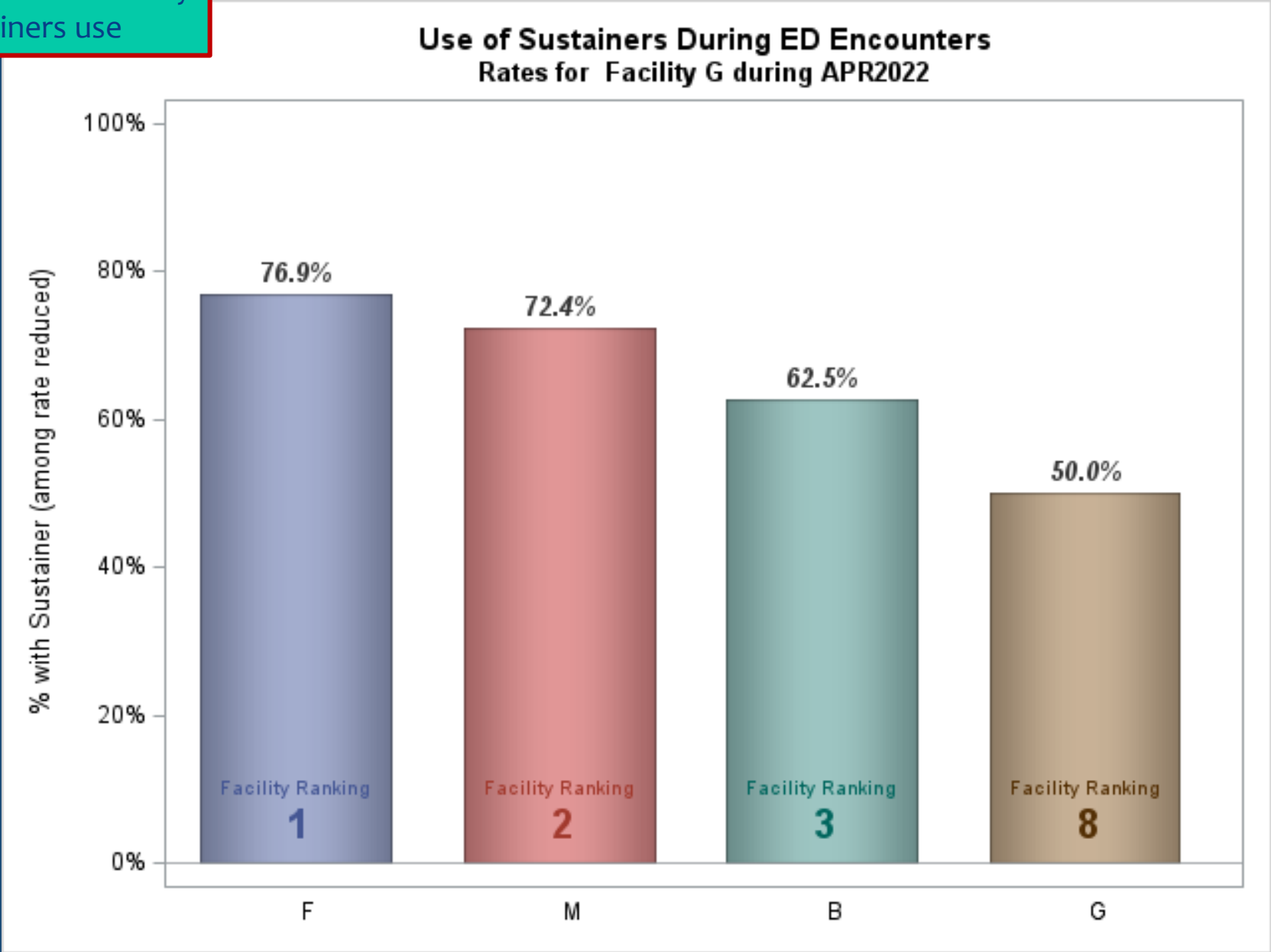

Supplement: Supplementary file 20 — Additional file 20. Example of facility-specific monthly graphic on sustainers use. [file 13063_2023_7230_MOESM20_ESM.pdf]
