# Supplementary material for: Clinical decision support to Optimize Care of patients with Atrial Fibrillation or flutter in the Emergency department: protocol of a stepped-wedge cluster randomized pragmatic trial (O’CAFÉ trial)
Source: Trials. 2023 Mar 31;24:246. doi: 10.1186/s13063-023-07230-2 (PMC10064588; doi:10.1186/s13063-023-07230-2)
Supplement: Supplementary file 4 — Additional file 4. Start screen. [file 13063_2023_7230_MOESM4_ESM.pdf]

Additional file 4:
Start Screen

Start Patient CHA_2DS_2-VASc Modules AC Recommendation Wrap-up Summary

LINCOLN, ABRAHAM • M0000000 • 211 Y M

ELIGIBILITY CRITERIA

Adults (≥18y) w/ AF or AFL who need rate control, stroke risk assessment, consideration for cardioversion, or are newly diagnosed with AFF today

- Confirmed with ECG (12-lead or monitor)
- Applicable if AFF is the primary dx; if secondary, first stabilize the primary dx

EXCLUDE

- Pregnancy
- STEMI
- Acute myocarditis or pericarditis
- Acute pulmonary embolism
- Shock (e.g., septic, hemorrhagic, cardiogenic)
- Major thoracic trauma (<48h)
- Thyroid storm
- Acute toxidrome (e.g., sympathomimetic or anticholinergic)
