# Supplementary material for: Clinical decision support to Optimize Care of patients with Atrial Fibrillation or flutter in the Emergency department: protocol of a stepped-wedge cluster randomized pragmatic trial (O’CAFÉ trial)
Source: Trials. 2023 Mar 31;24:246. doi: 10.1186/s13063-023-07230-2 (PMC10064588; doi:10.1186/s13063-023-07230-2)
Supplement: Supplementary file 5 — Additional file 5. Patient screen. [file 13063_2023_7230_MOESM5_ESM.pdf]

Additional file 5:
Patient Screen

Start | Patient | Triggers | CHA_2DS_2-VASc | Modules | AC Recommendation | Wrap-up | Summary

[TRAINING MODE]

PATIENT

Today

Rhythm — AF ◉ AFL ○ Both ○

On anticoagulant (po or subcutaneous) — Yes ○ No ◉

Cardiovascularly unstable — Yes ○ No ◉

> Acute ischemia, hypotension, or mod-to-severe pulmonary edema

Duration

Paroxysmal (<7d) ◉

Persistent (≥7d<1y) ○

Paroxysmal or persistent (#wks?) ○

Long-standing or permanent (>1y) ○

[illegible]nclear ○

[illegible] onset <48h, use slider: N/A

> Consider ordering a TSH if:
> - AFF is a new dx or
> - Known AFF but no recent TSH (e.g., within 6m)

History*

AF or AFL — [illegible]s ◉ No ○

Last TSH (normal range: 0.4 – 5.5 mIU/mL) — No TSH lab available

Last echocardiogram — EF 65% (2019-02-19)

> Consider ordering if AFF is a new dx and no echo <2y; also if new significant cardiac dysfxn or HF inconsistent with prior EF. Timing (outpt vs ED/inpt) depends on urgency.
