# Supplementary material for: Clinical decision support to Optimize Care of patients with Atrial Fibrillation or flutter in the Emergency department: protocol of a stepped-wedge cluster randomized pragmatic trial (O’CAFÉ trial)
Source: Trials. 2023 Mar 31;24:246. doi: 10.1186/s13063-023-07230-2 (PMC10064588; doi:10.1186/s13063-023-07230-2)
Supplement: Supplementary file 6 — Additional file 6. Trigger screen with explanation paragraph. [file 13063_2023_7230_MOESM6_ESM.pdf]

Additional file 6:
Trigger screen with explanation paragraph

Trigger screen pops up only for pts with **intermittent AFF** (paroxysmal or persistent).

Start — Patient — **Triggers** — CHA_2DS_2-VASc — Summary

TRIGGERS

Did you ask patient about the following under-reported treatable triggers of AFF?

- ○ Yes, I asked patient about cold food, cold drink, and/or alcohol
- ○ No, did not discuss w/patient
- ○ Patient unable to reply (AMS, ETT, etc)

Does the patient report the following triggers?

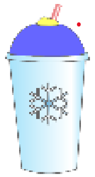

Cold drink/food immediately prior to experiencing AFF*

Current episode	Yes ○	No ○
History of prior episodes	Yes ○	No ○

Pts who are + for cold-induced AFF may be contacted by CREST to participate in a survey study

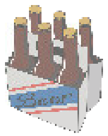

Alcohol (light or heavy)†

Current episode	Yes ○	No ○
History of prior episodes	Yes ○	No ○

* E.g., ice-cold drink, slushie, ice cream; occurs within seconds to minutes of ingestion (cf. Vinson. Cold Drink Heart. *Perm J.* 2020;24:19.238).

† Alcohol exposure can be light (as little as a drink within the prior 4 hours) or heavy (in which AFF develops during or within 36 hours of a binge). See references for more info.

Additional file 6: Supplemental text for Additional file 6

Text to supplement image of Additional file 5.

Addressing Common Reversible Dietary Triggers

Though patients with symptomatic AFF commonly seek medical care in the ED, little attention in emergency medicine has been paid to the clinician's role in helping patients identify and manage reversible triggers of paroxysmal AFF. Our CDSS prompts clinicians to ask patients with paroxysmal or persistent AFF about two common ingestion triggers: cold drink/food and alcohol.

Cold drink and food can precipitate AF within seconds or minutes of ingestion and occasionally co-occur with "brain freeze".[1] A prevalent AFF trigger, cold ingestions are often unacknowledged by clinicians or discounted altogether when patients report an association.[2] Alcohol is known to precipitate AF during or following an alcohol binge.[3] Evidence demonstrates that AF also can develop within several hours of even one or two drinks.[4]

When inquiries about these two ingestion triggers elicit a positive response, the stage is set for the clinician to suggest behavioral changes that may reduce recurrence and decrease a patient's AFF burden.[5] A simple change in diet can reduce the number of AFF episodes (and their distressing symptoms and associated health care risk), along with a reduction in the inconvenience and cost of missed days of work and the need for urgent medical care.[6] We omitted coffee consumption from our list of dietary AFF triggers because the evidence does not support the commonly held belief that coffee triggers AFF.[7, 8]
