# Supplementary material for: Clinical decision support to Optimize Care of patients with Atrial Fibrillation or flutter in the Emergency department: protocol of a stepped-wedge cluster randomized pragmatic trial (O’CAFÉ trial)
Source: Trials. 2023 Mar 31;24:246. doi: 10.1186/s13063-023-07230-2 (PMC10064588; doi:10.1186/s13063-023-07230-2)

Additional file 7: CHA_2DS_2 Screen

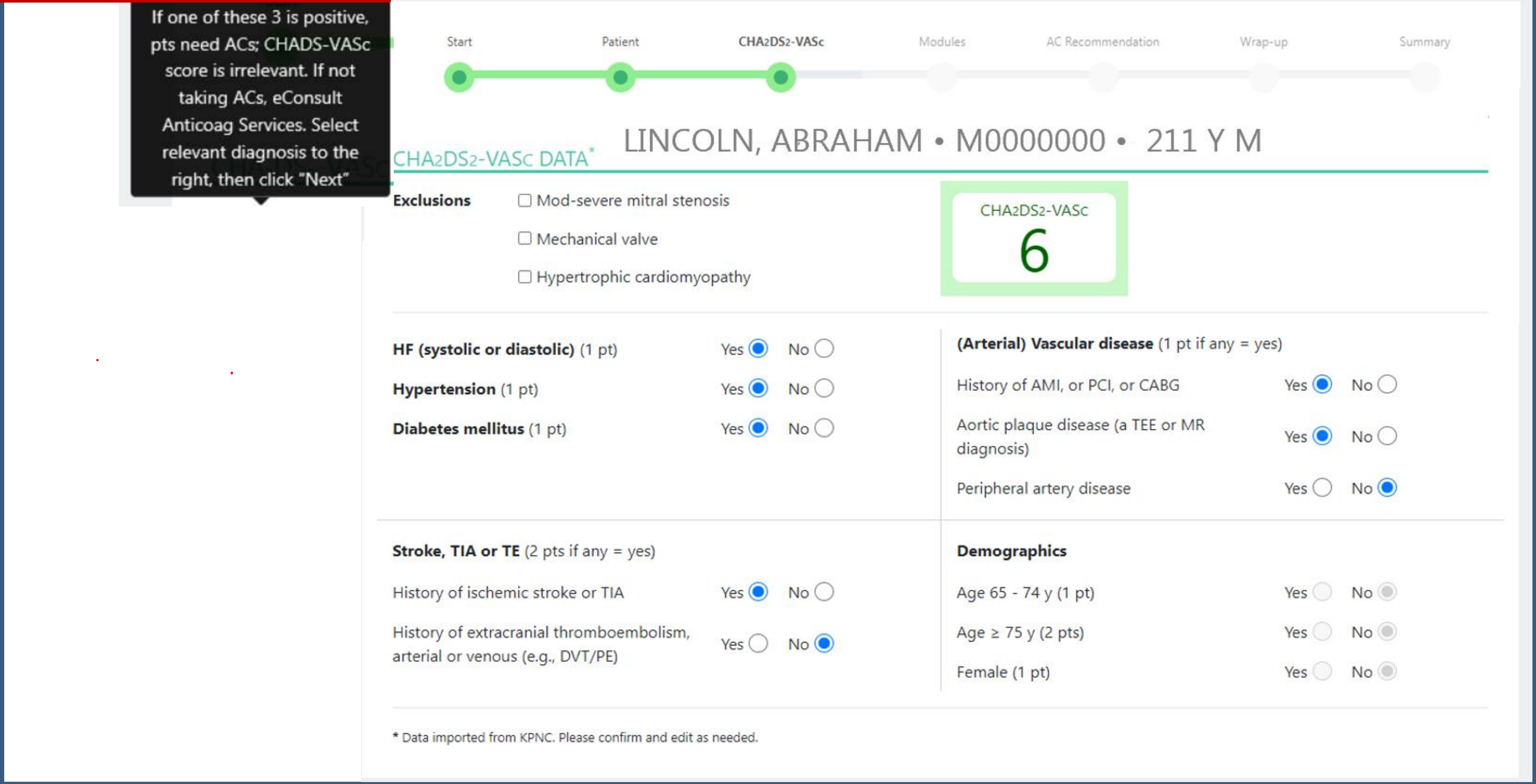

Supplement: Supplementary file 7 — Additional file 7. CHA2DS2-VASc Screen. [file 13063_2023_7230_MOESM7_ESM.pdf]
