# Supplementary material for: Clinical decision support to Optimize Care of patients with Atrial Fibrillation or flutter in the Emergency department: protocol of a stepped-wedge cluster randomized pragmatic trial (O’CAFÉ trial)
Source: Trials. 2023 Mar 31;24:246. doi: 10.1186/s13063-023-07230-2 (PMC10064588; doi:10.1186/s13063-023-07230-2)
Supplement: Supplementary file 8 — Additional file 8. Rate control for a weak heart. [file 13063_2023_7230_MOESM8_ESM.pdf]

Additional file 8:
Rate control for a weak heart

RATE CONTROL Weak ♥

Low EF (≤50%) or decompensated HF

Engage cardiology early. These pts often need hospitalization.

- Prioritize HF treatment (using vasodilators, diuretics, etc.)
- Avoid non-dihydropyridine calcium channel blockers (CCBs), like dilt and verapamil
- If SBP low or volume overloaded, avoid beta-blockers (BBs), too
- If unstable or with pre-excitation (WPW), consider DCCV
  - In HFrEF, however, cardioversion is often poorly effective and not very durable

Non-Beta-blocker Options*

- IV amiodarone: ± loading dose, then maintenance†
- Or IV digoxin

Beta-blockers

If the pt has stabilized, may cautiously try BBs to reduce RVR (common target 110-120)

- IV metoprolol 2.5-5.0mg over 2m; may repeat q10-15m if SBP stable; up to 10-15mg total
- IV esmolol infusion* may be safer than metoprolol as esmolol is more titratable

* See "AF Order Set" for amiodarone, digoxin, and esmolol doses
† Rate reduction effect of amiodarone is not delayed like its rhythm effect
