# Supplementary material for: Clinical decision support to Optimize Care of patients with Atrial Fibrillation or flutter in the Emergency department: protocol of a stepped-wedge cluster randomized pragmatic trial (O’CAFÉ trial)
Source: Trials. 2023 Mar 31;24:246. doi: 10.1186/s13063-023-07230-2 (PMC10064588; doi:10.1186/s13063-023-07230-2)
Supplement: Supplementary file 9 — Additional file 9. Rate control if cardioverting. [file 13063_2023_7230_MOESM9_ESM.pdf]

Additional file 9:
Rate control if cardioverting

SELECTIVE RATE CONTROL if Cardioversion Intended

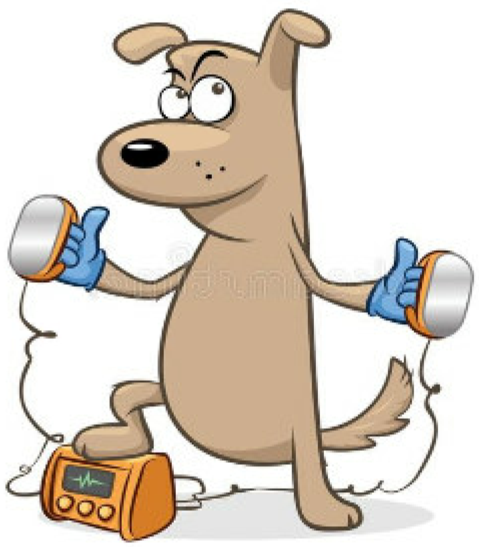

May *forgo* IV rate reduction (RR)

- If cardioverting soon and pt tolerating RVR
- Exception: RR meds recommended ≥30 min before Class IC agents (oral flecainide and propafenone)

IV RR meds are *not* entirely benign

- Increase risk of hypotension and post-cardioversion bradycardia
- May reduce the effectiveness of cardioversion (*cf. Blecher. CJEM. 2012.*)

If indicated, use IV bolus RR meds when:

- Not tolerating RVR and cardioversion delayed
  - Or if RVR consistently ≥160
- Use IV metoprolol or IV dilt: see “AF order set” for doses
