# Supplementary material for: Clinical decision support to Optimize Care of patients with Atrial Fibrillation or flutter in the Emergency department: protocol of a stepped-wedge cluster randomized pragmatic trial (O’CAFÉ trial)
Source: Trials. 2023 Mar 31;24:246. doi: 10.1186/s13063-023-07230-2 (PMC10064588; doi:10.1186/s13063-023-07230-2)
Supplement: Supplementary file 10 — Additional file 10. Eligibility for ED cardioversion. [file 13063_2023_7230_MOESM10_ESM.pdf]

Additional file 10:
Eligibility for ED cardioversion

WHO IS ELIGIBLE?

Emergent Cardioversion

Use DCCV for

a. Significant hemodynamic instability, or
b. Ongoing myocardial ischemia, hypotension, or HF from RVR not responding promptly to meds, or
c. Pre-excitation, like WPW

Requires anticoagulation

Elective Cardioversion

Must be (a) clearly recent-onset AFF (<48h), or (b) recent TEE (<48h) negative for thrombus, or (c) paroxysmal or persistent AFF <4w and adequately anticoagulated for at least 3w. Contraindicated in digoxin toxicity or K^+ <3.5mEq/L, or mechanical valve w/o adequate pre-ED anticoagulation.

- **Best candidates** have one or more of the following characteristics:
  - First AFF episode or infrequent episodes
  - Markedly symptomatic AFF (usually RVR)
  - Structurally normal heart
  - Younger, healthier patient
  - Low CHA_2DS_2-VASc (0-1)
  - Patient preference
- **Advantages**
  - AFF symptom resolution
  - Reduces need for hospitalization
  - Reduces electrical and structural remodeling from persistent AFF
- **Address other AFF medication needs**
  - Antecedent IV **rate reduction medications may reduce effectiveness** of cardioversion efforts (cf. Blecher. *CJEM*. 2012.)
  - **If CHA_2DS_2-VASc score $\geq 2_m$ or $\geq 3_f$** anticoagulate in ED and at least 4w post
