# Supplementary material for: Clinical decision support to Optimize Care of patients with Atrial Fibrillation or flutter in the Emergency department: protocol of a stepped-wedge cluster randomized pragmatic trial (O’CAFÉ trial)
Source: Trials. 2023 Mar 31;24:246. doi: 10.1186/s13063-023-07230-2 (PMC10064588; doi:10.1186/s13063-023-07230-2)
Supplement: Supplementary file 11 — Additional file 11. Debate over timing of ED elective cardioversion. [file 13063_2023_7230_MOESM11_ESM.pdf]

Additional file 11:
Debate over timing of ED elective cardioversion

When: Today or Tomorrow?

Short-term Delayed Cardioversion?

- Some AF pts who present early after sx-onset (<24h) and are candidates for elective ED cardioversion might be eligible to go home, deferring cardioversion till scheduled follow-up at 36-40h from sx-onset.
- This requires shared decision-making about pros and cons (below).
- If all agree, achieve ED rate control (avg RVR<110), address stroke risk based on CHA_2DS_2-VASc score, then have the pt return to ED at 36-40h from sx-onset (in the Netherland trial by Pluymaekers, pts followed-up first in the clinic, then ED only if needed; this is a more cumbersome approach).

Pros of Short-term Delay

- 2/3 spontaneously cardiovert <48h of sx-onset
- This avoids the risks a/w physician-initiated cardioversion
- Timing of cardioversion (today vs tomorrow) does not affect incidence of sinus rhythm at 4w (about 70%) or quality-of-life scores

Cons of Short-term Delay

- Requires a return visit to identify the 1/3 with continued AF
- Additional visits are inconvenient and costly (e.g., co-pays)
- The next doc might disagree with the plan, as we found in a prior KP study (cf. Vinson. *J Emerg Med*. 2012).
- Early cardioversion increases pt satisfaction with the ED visit (cf. Ballard. *Ann Emerg Med*. 2015).
- Some data suggest that cardioversion performed <12h is safer than that performed 12h-48h from onset (cf. Nuotio. *JAMA*. 2014).
