# Supplementary material for: Clinical decision support to Optimize Care of patients with Atrial Fibrillation or flutter in the Emergency department: protocol of a stepped-wedge cluster randomized pragmatic trial (O’CAFÉ trial)
Source: Trials. 2023 Mar 31;24:246. doi: 10.1186/s13063-023-07230-2 (PMC10064588; doi:10.1186/s13063-023-07230-2)
Supplement: Supplementary file 12 — Additional file 12. Anticoagulation when cardioverting. [file 13063_2023_7230_MOESM12_ESM.pdf]

Additional file 12: Anticoagulation when cardioverting

ANTICOAGULATE?

When you cardiovert pts

Population: ED pts with non-valvular* AFF, not anticoagulated, eligible for ED cardioversion (i.e., AFF clearly <48h, or TEE neg <48h, or needing emergent cardioversion†)

Low Stroke Risk

CHA_2DS_2-VASc score $\leq 1_m$ or $\leq 2_f$

AHA Guidelines (2014/2019; section 6.1.1)

- Pre-OAC for 3w not needed with this population
- ED anticoagulation "may be considered": before (IV or sq) or immediately after (IV, sq or po) cardioversion
- Post-cardioversion OAC for 4w not needed

The Canadian Guidelines recommend AC for some low-risk patients
Many TPMG Cardiologists advise adding 4w of post-cardioversion OAC following physician-led cardioversion‡ (link to AC recommendation)

High Stroke Risk

CHA_2DS_2-VASc score $\geq 2_m$ or $\geq 3_f$

AHA Guidelines (2014/2019; section 6.1.1)

- Pre-OAC for 3w not needed
- ED anticoagulation "recommended": AC before (IV or sq) or immediately after (IV, sq or po) cardioversion
- Post-cardioversion OAC required for at least 4w (link to risk-specific stroke prevention screen)

* Thrombogenic valve disease = moderate-to-severe mitral stenosis, any mechanical valve, or HCM. If such a pt is not already on OACs (and they should be) yet undergoes ED cardioversion, consider ED LMWH and consult Anticoag Services or cardiology for advice on long-term OAC.

† For emergent cardioversion (i.e., for unstable pts), the AHA and CHEST (ACCP) Guidelines recommend a pre-cardioversion dose of AC in the ED (if possible) and 4w of post-cardioversion ACs, regardless of risk score.

‡ For an evidence-based rationale, see Andrade. *Can J Cardiol*. 2019.
