# Supplementary material for: Clinical decision support to Optimize Care of patients with Atrial Fibrillation or flutter in the Emergency department: protocol of a stepped-wedge cluster randomized pragmatic trial (O’CAFÉ trial)
Source: Trials. 2023 Mar 31;24:246. doi: 10.1186/s13063-023-07230-2 (PMC10064588; doi:10.1186/s13063-023-07230-2)
Supplement: Supplementary file 13 — Additional file 13. Ibutilide decision aid. [file 13063_2023_7230_MOESM13_ESM.pdf]

Additional file 13:
Ibutilide decision aid

IBUTILIDE CLINICAL AID

Advantages

- Rapid effect for AF and AFL
- Hemodynamically neutral
- Drug-of-choice for **AFL** (80% effective by 4h)

To ↓ Risk of VT

- Avoid with structural heart disease
- Confirm normal serum K^+ and Mg^+
- Confirm QTc interval <480 ms
- ECG monitoring for 4h post

Administration

Weight-based Dosing

- If weight >60 kg, 1 mg over 10m
- If weight <60 kg, use 0.01 mg/kg

Give Both Half-doses

After 10m interval, give 2nd half-dose unless the 1st results in:

- Cardioversion
- Adverse events, e.g. VT
- QTc prolongation >480 ms

1st half-dose sufficient for DCCV pre-treatment (to ↑ DCCV effect)

Allow at least 60m for effect

Optional Adjuncts

$MgSO_4$ Supplement

- To ↑ ibutilide effectiveness 3-4g over 1h pre-ibutilide (supported by several studies)
- To ↓ incidence of VT 5g over 1h pre-ibutilide <u>and</u> 5g over 2h during/post (supported by one trial)

Source: Adapted from Fig E2 in Vinson. *Ann Emerg Med.* 2018.
