# Supplementary material for: Clinical decision support to Optimize Care of patients with Atrial Fibrillation or flutter in the Emergency department: protocol of a stepped-wedge cluster randomized pragmatic trial (O’CAFÉ trial)
Source: Trials. 2023 Mar 31;24:246. doi: 10.1186/s13063-023-07230-2 (PMC10064588; doi:10.1186/s13063-023-07230-2)

Additional file 13:
Anticoagulation recommendations for patients with high estimated annual risk for ischemic stroke

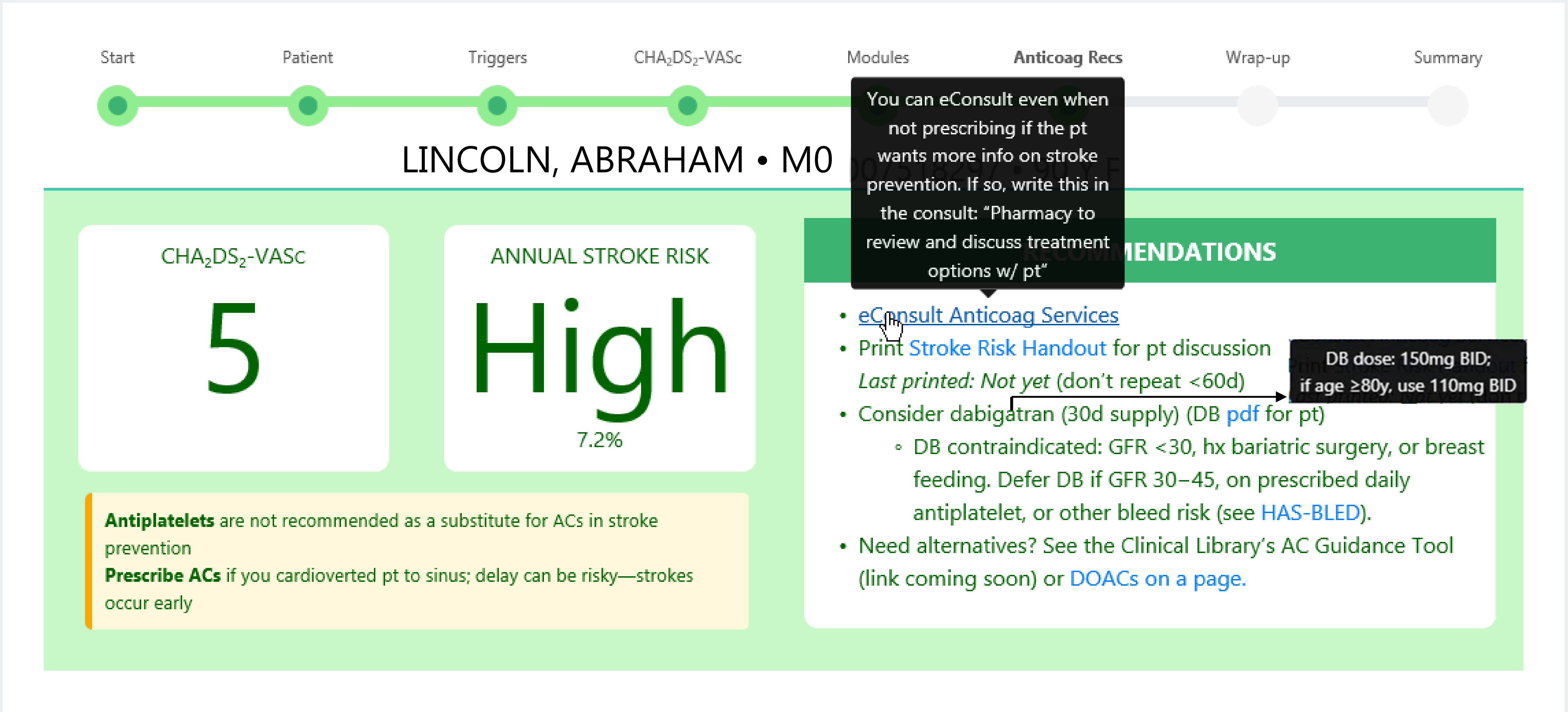

Supplement: Supplementary file 14 — Additional file 14. Anticoagulation recommendations for patients with high estimated annual risk for ischemic stroke. [file 13063_2023_7230_MOESM14_ESM.pdf]
