# Supplementary material for: Clinical decision support to Optimize Care of patients with Atrial Fibrillation or flutter in the Emergency department: protocol of a stepped-wedge cluster randomized pragmatic trial (O’CAFÉ trial)
Source: Trials. 2023 Mar 31;24:246. doi: 10.1186/s13063-023-07230-2 (PMC10064588; doi:10.1186/s13063-023-07230-2)
Supplement: Supplementary file 15 — Additional file 15. Patient handout on stroke risk. [file 13063_2023_7230_MOESM15_ESM.pdf]

Additional file 15:
Patient handout on stroke risk

Atrial Fibrillation (A Fib), Atrial Flutter (A Flutter), and your Risk of Stroke

Prepared for: ______________________________

1 Your A Fib/Flutter Diagnosis

- The irregular heartbeat of A Fib/Flutter allows small blood clots to form in the heart. Clots can travel to the brain, block blood flow, and cause a stroke.
- A stroke can cause sudden numbness or weakness of the face, arm, or leg, especially on one side of the body. Strokes can also cause sudden confusion, trouble speaking or understanding, and even trouble seeing in one or both eyes. Strokes can cause chronic disability and even death.
- A Fib and A Flutter increase your risk for stroke and death. But not everyone with A Fib/Flutter has the same risk. We calculated your personal stroke risk based on your age, sex, and medical conditions.[1]

2 Reducing Your Stroke Risk

- If your annual stroke risk crosses a threshold, medications called anticoagulants are usually recommended to reduce your risk of stroke (and clots in other places, too).
- Common anticoagulants include dabigatran, warfarin, and rivaroxaban.

3 Your Personal Stroke Risk Evaluation

Your risk of having a stroke can be estimated by comparing you to people with A Fib or Flutter who have similar age, sex, and medical conditions as yourself.[1]

Of every **100** people like you, **7** will have a stroke over the next year if not treated

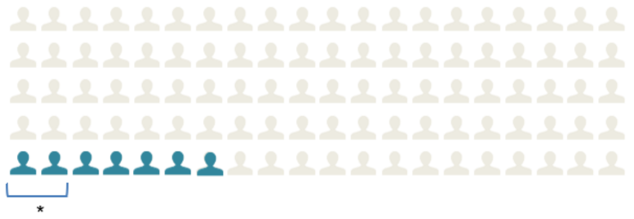

Anticoagulants reduce

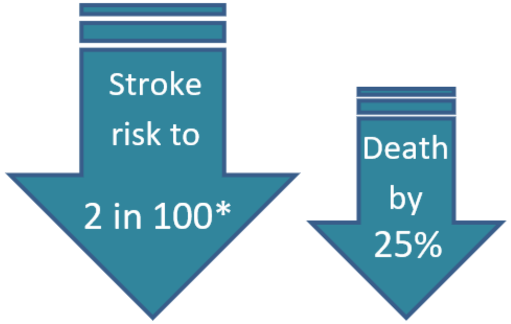

4 Reducing Risk of Bleeding when Taking Anticoagulants

Anticoagulants work by reducing your ability to clot. This can increase the risk of bleeding, even in the brain, though this is rare (~0.5% annually [1 in 200]).

You can reduce the chance of a having a bleeding complication from anticoagulants in these ways:

- Avoid taking aspirin unless prescribed
- Avoid taking non-steroidal anti-inflammatory medications like ibuprofen (Advil) and naproxen (Aleve)
- If you have high blood pressure (hypertension), keep your blood pressure well controlled
- Avoid excess alcohol (8 or more drinks per week)
- Report any new symptoms to your primary care provider

5 Next Steps

- Learn more about the benefits and risks associated with stroke prevention medications by talking with your primary care provider
- Discuss this handout at your next appointment
