# Supplementary material for: Clinical decision support to Optimize Care of patients with Atrial Fibrillation or flutter in the Emergency department: protocol of a stepped-wedge cluster randomized pragmatic trial (O’CAFÉ trial)
Source: Trials. 2023 Mar 31;24:246. doi: 10.1186/s13063-023-07230-2 (PMC10064588; doi:10.1186/s13063-023-07230-2)
Supplement: Supplementary file 16 — Additional file 16. About HAS-BLED. [file 13063_2023_7230_MOESM16_ESM.pdf]

Additional file 16:
About HAS-BLED

HAS-BLED

ABOUT | CONDITIONS

HAS-BLED is an Outpatient Tool to Help Reduce Bleeding Risk

- Its fundamental purpose is to draw attention to reversible risk factors that need correcting
- Pts with a higher risk of bleeding (e.g., score ≥3) require more careful review by the outpt care team and closer monitoring
- These variables are modifiable:
  - Inadequate blood pressure control
  - Inappropriate medication use (e.g., NSAIDs) while on OACs
  - Excess alcohol use
- An elevated score should not be an excuse to withhold OAC, as pts at higher risk for bleeding complications are those who benefit even MORE from stroke prevention
- Studies show that pts place greater value on stroke prevention than bleeding avoidance

---

Cf. Lip. *J Thromb Haemost*. 2016.

(Greg Lip is the originator of the CHA_2DS_2-VASc and HAS-BLED scores.)
