# Supplementary material for: Clinical decision support to Optimize Care of patients with Atrial Fibrillation or flutter in the Emergency department: protocol of a stepped-wedge cluster randomized pragmatic trial (O’CAFÉ trial)
Source: Trials. 2023 Mar 31;24:246. doi: 10.1186/s13063-023-07230-2 (PMC10064588; doi:10.1186/s13063-023-07230-2)
Supplement: Supplementary file 17 — Additional file 17. HAS-BLED score and risk. [file 13063_2023_7230_MOESM17_ESM.pdf]

Additional file 17:
HAS-BLED Score and Risk

HAS-BLED

ℹ ABOUT   CONDITIONS

Condition*	Points
**H**ypertension, uncontrolled	1
**A**bnormal renal or liver function (1 each)	1 or 2
**S**troke history (ischemic or hemorrhagic)	1
**B**leeding history or anemia or low platelets	1
**L**abile INR	1
**E**lderly (>65 years)	1
**D**rugs (antiplatelets) or alcohol (1 each)	1 or 2

HAS-BLED Score	Annual Bleed Risk†	Annual Risk Group
0	1.1%	
1	1.0%	Low
2	1.9%	
3	3.8%	
4	8.7%	High
≥5	12.5%	

* Recent definitions found in Hindricks. *Eur Heart J.* 2020 guidelines.
† Pisters. *CHEST.* 2010
