# Supplementary material for: Clinical decision support to Optimize Care of patients with Atrial Fibrillation or flutter in the Emergency department: protocol of a stepped-wedge cluster randomized pragmatic trial (O’CAFÉ trial)
Source: Trials. 2023 Mar 31;24:246. doi: 10.1186/s13063-023-07230-2 (PMC10064588; doi:10.1186/s13063-023-07230-2)
Supplement: Supplementary file 18 — Additional file 18. Wrap-up screen [file 13063_2023_7230_MOESM18_ESM.pdf]

Additional file 18:
Wrap-up screen

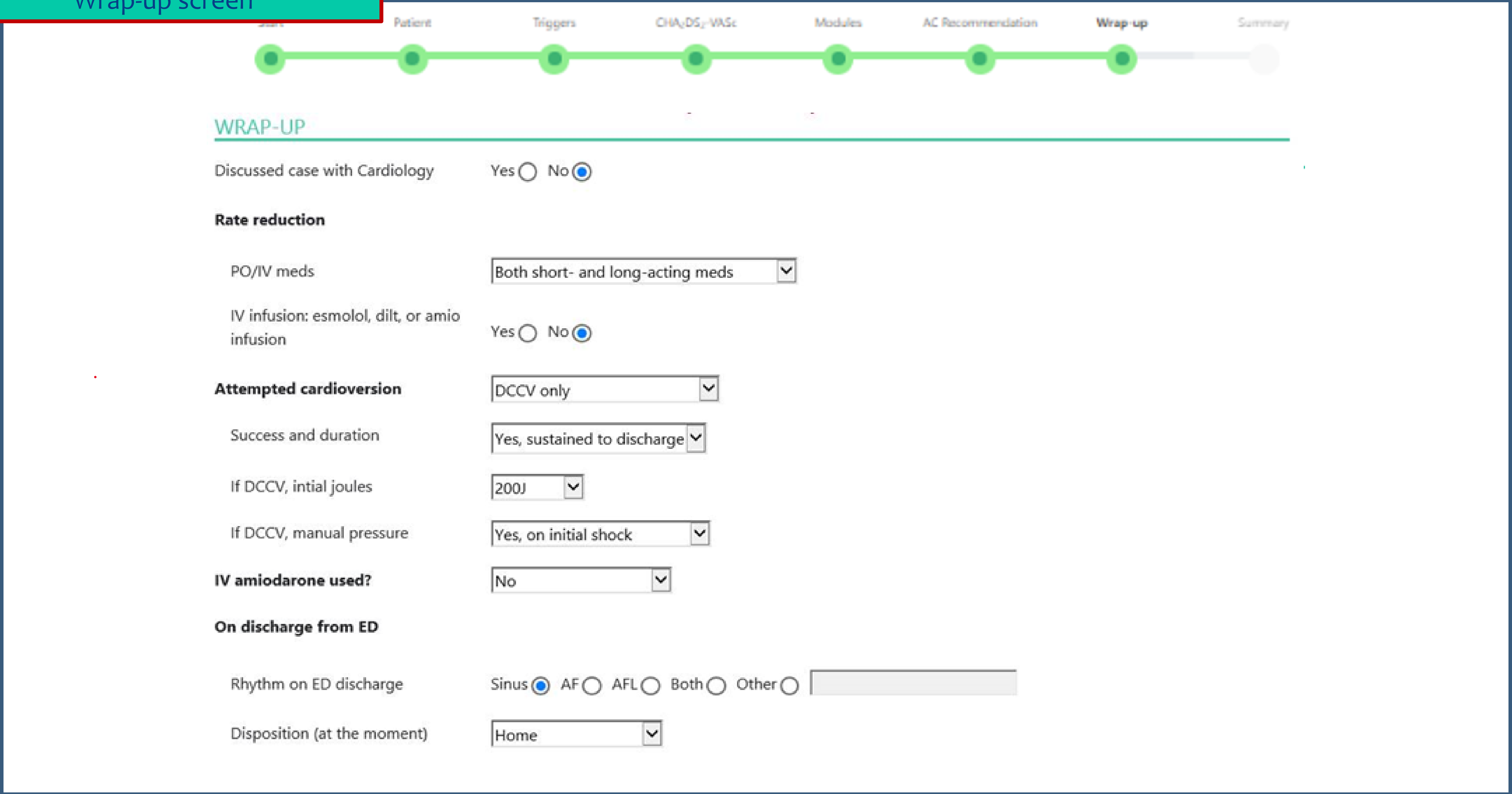

Patient | Triggers | CHA_2DS_2-VASc | Modules | AC Recommendation | **Wrap-up** | Summary

WRAP-UP

Discussed case with Cardiology — Yes ○ No ◉

Rate reduction

PO/IV meds — Both short- and long-acting meds

IV infusion: esmolol, dilt, or amio infusion — Yes ○ No ◉

Attempted cardioversion — DCCV only

Success and duration — Yes, sustained to discharge

If DCCV, intial joules — 200J

If DCCV, manual pressure — Yes, on initial shock

IV amiodarone used? — No

On discharge from ED

Rhythm on ED discharge — Sinus ◉ AF ○ AFL ○ Both ○ Other ○

Disposition (at the moment) — Home
