# Supplementary material for: Clinical decision support to Optimize Care of patients with Atrial Fibrillation or flutter in the Emergency department: protocol of a stepped-wedge cluster randomized pragmatic trial (O’CAFÉ trial)
Source: Trials. 2023 Mar 31;24:246. doi: 10.1186/s13063-023-07230-2 (PMC10064588; doi:10.1186/s13063-023-07230-2)
Supplement: Supplementary file 19 — Additional file 19. Summary to paste into physician charting. [file 13063_2023_7230_MOESM19_ESM.pdf]

Additional file 19:
Summary to paste into physician charting

Start · Patient · Triggers · CHA_2DS_2-VASc · Modules · AC Recommendation · Wrap-up · **Summary**

LINCOLN, ABRAHAM • M0000000 • 211 Y M

SUMMARY

Regarding LINCOLN, ABRAHAM'S AF/AFL history and management:

He does not have a known history of prior A Fib or Flutter. Today's rhythm is A Fib, which seems to be paroxysmal in nature. Onset was approximately 12 hours prior to arrival. Prior to presentation, no TSH was documented.

The patient had a rapid ventricular response. We provided rate reduction with short-acting IV medications (see orders for details). He did not require a continuous infusion of AV nodal blockers. We attempted sinus restoration with electrical cardioversion. See procedure note for details of DCCV. Cardioversion was successful and sustained to discharge.

His CHA_2DS_2-VASc score during this encounter was 5 points, which placed him in a high-risk category for stroke. There is also a slightly elevated risk of short-term stroke following ED cardioversion, regardless of CHA_2DS_2-VASc score.

We provided a dose of anticoagulants in the ED. I printed the risk-specific stroke prevention patient handout, discussed it with the patient (or representative) and encouraged him to review it with his primary care provider. An e-consult was submitted to the Anticoagulation Services for follow-up. I prescribed anticoagulants for discharge after discussing the risks and benefits with the patient (or representative). I gave him the KP handout on the anticoagulant.

At the time of ED discharge, he was in sinus rhythm. Current disposition plan is to home.
